# Importance of benzoyltransferase GcnE and lysine benzoylation of alcohol dehydrogenase AdhB in pathogenesis and aflatoxin production in *Aspergillus flavus*

**DOI:** 10.1128/mbio.02665-24

**Published:** 2024-11-27

**Authors:** Xuan Chen, Lihan Wu, Yuqi Zhang, Sen Wang, Shihua Wang

**Affiliations:** 1 State Key Laboratory of Ecological Pest Control for Fujian and Taiwan Crops, Key Laboratory of Pathogenic Fungi and Mycotoxins of Fujian Province, and School of Life Sciences, Fujian Agriculture and Forestry University, Fuzhou, China; Duke University School of Medicine, Durham, North Carolina, USA

**Keywords:** benzoylation, *Aspergillus flavus*, alcohol dehydrogenase, GcnE, aflatoxin

## Abstract

Lysine benzoylation (Kbz) is a newly identified post-translational modification associated with active transcription and metabolism in eukaryotes. However, whether Kbz exists in pathogenic fungi and its function remains unknown. Here, we demonstrated for the first time that Kbz is present in *Aspergillus flavus* and identified 60 benzoylated sites on 46 benzoylated proteins by global benzoylome analysis. Our data demonstrated that alcohol dehydrogenase B (AdhB) is regulated by benzoylation on lysine 321 (K321), and mutations of Kbz site in AdhB significantly reduced the alcohol dehydrogenase activity *in vivo* and *in vitro*. Both *adhB* deletion mutant and benzoylated site mutants (K321R and K321A) exhibited similar phenotype, including decreased conidiation and seed colonization, increased sclerotia formation and aflatoxin production, and more sensitive to cell wall damage stress. We also found that GcnE has benzoyltransferase activity *in vitro* and *in vivo*, and its repression leads to decreased Kbz level and enzymatic activity of AdhB. The catalytic site E139 is important for the benzoyltransferase function of GcnE. Our study uncovers a previously unknown mechanism by which benzoylation regulates AdhB activity to affect the development, secondary metabolism, pathogenicity, and stress response of *A. flavus*. Meanwhile, it points out the important role of Kbz in the pathogenicity of pathogenic fungi.

**IMPORTANCE:**

*Aspergillus flavus* is a ubiquitous opportunistic pathogen of plants and animals, which produces carcinogenic and toxic secondary metabolite aflatoxin. *A. flavus* and aflatoxin contamination have emerged as a global food safety concern. Currently, post-translational modification plays crucial modulatory roles in the fungal development and virulence, but the role of benzoylation in fungal pathogenicity remains undetermined, which limits the development of prevention and control technique. Here, we first identified 46 benzoylated proteins in *A. flavus*, and found that benzoyltransferase GcnE exerted effects on pathogenicity and aflatoxin production by regulating the benzoylation of AdhB. This finding not only provided valuable information for prevention and control of *A. flavus* contamination, but also offered basic knowledge for investigation of the regulation mechanism of secondary metabolism in other fungi.

## INTRODUCTION

Protein post-translational modifications (PTMs) play a critical role in various biological processes by regulating protein function (1). An increasing number of PTMs have been identified via mass spectrometry (MS) and pan-antibody enrichment techniques, and lysine acylation is one of the most abundant and well-studied PTMs, which widely detected in both histone and non-histone proteins (1). Up to now, fourteen acylation members were identified, including formylation (1), acetylation (1), propionylation (1), butyrylation (1), 2-hydroxyisobutyrylation (1), β-hydroxybutyrylation (1), crotonylation (1), malonylation (1), succinylation (1), glutarylation (1), benzoylation (2), lactylation (3), isonicotinylation (4), and methacrylation (5). Meanwhile, numerous studies have proved that acylation plays an important role in fungal virulence and pathogenic morphogenesis (6, 7). For example, the acetylomes of the three deadliest human fungal pathogens, *Cryptococcus neoformans*, *Candida albicans*, and *Aspergillus fumigatus* show that the level of acetylation in pathogenic fungi correlates with their pathogenicity (7).

Lysine benzoylation (Kbz) is a relatively new PTM originally identified in histone (2), which was structurally similar to acetylation (Kac). More notably, Kbz has a benzene ring group, and it not only neutralizes the positive charge of lysine, but is also able to induce a larger size change and stronger hydrophobicity compared to Kac. Likewise, histone Kbz appears to be consistent with acetylation, associated with active gene transcription and some metabolism (2). At present, Kbz is only found in yeast, RAW cells, and HepG2 cells, and 27, 18, and 18 histone benzoylated sites were identified, respectively (2, 8). Meanwhile, deacylase Sirt2 can remove benzoylated group from histone lysine, and acyltransferases GcnE and EsaA have histone benzoyltransferase activity in yeast (2, 8). Consistent with other acylation, benzoylation has multiple substrates including histone acylation sites as well as non-histone proteins, and the benzoylated non-histone proteins were involved in diverse biological processes including glycolysis/gluconeogenesis, ribosome biogenesis, and others (8). However, the role of Kbz in pathogenic fungi remains unexplored.

*Aspergillus flavus* is a global pathogenic fungus, which is widely distributed in the natural environment (9). *A. flavus* not only causes Aspergillosis and fungal keratitis infections, but also colonizes pre- and post-harvest crops, then results in serious public health and dramatic economic losses to the agricultural industry (10). Meanwhile, *A. flavus* can produce abundant secondary metabolites, including series of mycotoxin, like aflatoxin, cyclopianilic acid, and aflatrem (11). Of these toxins, aflatoxin is the most abundant secondary metabolite in *A. flavus*. The most toxic form of aflatoxin is aflatoxin B_1_ (AFB_1_), which is one of the most toxic natural contaminants (9). Since aflatoxin is able to spread through the food chain, aflatoxin contamination of crops becomes a worldwide food safety concern (12). Considering the great harm of *A. flavus* and aflatoxin, it is necessary to study the regulatory mechanism of pathogenicity and clarify the regulatory network of aflatoxin biosynthesis in *A. flavus*.

Aflatoxin biosynthesis is regulated by complex mechanisms, and this regulation involves a complicated interaction among different PTMs (13). For example, MAPK pathway-related tyrosine phosphatases Msg5 and Yvh1 deletion resulted in significantly decreased aflatoxin production (14). Acetylation, succinylation, and 2-hydroxybutyrylation modifications occur in protein, which is located in aflatoxin biosynthesis cluster, and lead to alterations in aflatoxin production (15–17). Despite these results implying considerable roles of acylation in aflatoxin biosynthesis, the function of Kbz in aflatoxin biosynthesis remains unclear. Benzoate acts as an essential precursor substance, which is a necessary precondition for benzoylation. On the one hand, fungi can produce endogenous sodium benzoate through the β-oxidative and non-oxidative pathways of phenylalanine (18). On the other hand, exogenous addition of sodium benzoate can stimulate Kbz by generating benzoyl-CoA (2, 8), and sodium benzoate also inhibits the growth and aflatoxin production of *A. flavus* (19). Therefore, we speculate that benzoylation can regulate the growth and aflatoxin biosynthesis in *A. flavus*, but it requires further experiment to verify.

The purpose of this study was to explore the prevalence and biological functions of benzoylation in *A. flavus*. We performed high-resolution mass spectrometry to quantify benzoylated proteins and sites in *A. flavus*. Western blot (WB) and phenotypic assays showed that benzoylation defect of alcohol dehydrogenase B (AdhB) leads to reduced enzymatic activity and further affects development, aflatoxin biosynthesis, and pathogenicity. We also find that GcnE is a benzoyltransferase for AdhB. Therefore, we concluded that benzoylation plays key roles in fungal development, aflatoxin production and pathogenicity of *A. flavus*. This is the first functional study to reveal the role of Kbz in fungal virulence, and it provides a new insight into the prevention and control of fungal disease.

## RESULTS

### Lysine benzoylation is widely distributed among prokaryotes and eukaryotes

At present, benzoylation has been reported only in yeast and mammalian cells (2, 8), and increasing number of acylation has been shown to be evolutionarily conserved across species, such as animals, microorganisms, and plants (1). To assess whether the Kbz modification widely exists in prokaryotes and eukaryotes, we performed immunoblotting with a Kbz pan-antibody in *Escherichia coli*, *Saccharomyces cerevisiae*, *Arabidopsis*, *Danio rerio*, *Mus musculus*, and RAW cells. The results showed that Kbz signals were detected in all above species (Fig. S1A). Meanwhile, we detected Kbz modification in different fungi, including *A. flavus*, *A. fumigatus*, *A. nidulans*, and *S. cerevisiae*, and the results showed that Kbz was also detected in these fungi with different Kbz signals (Fig. S1B), these variations may be attributed to differences in culture conditions and potential evolutionary divergences in benzoylation between different fungus. All these results demonstrated that Kbz is widely distributed in prokaryotes and eukaryotes.

### Sodium benzoate inhibits the development and aflatoxin synthesis of *A. flavus*

**Fig 1:**
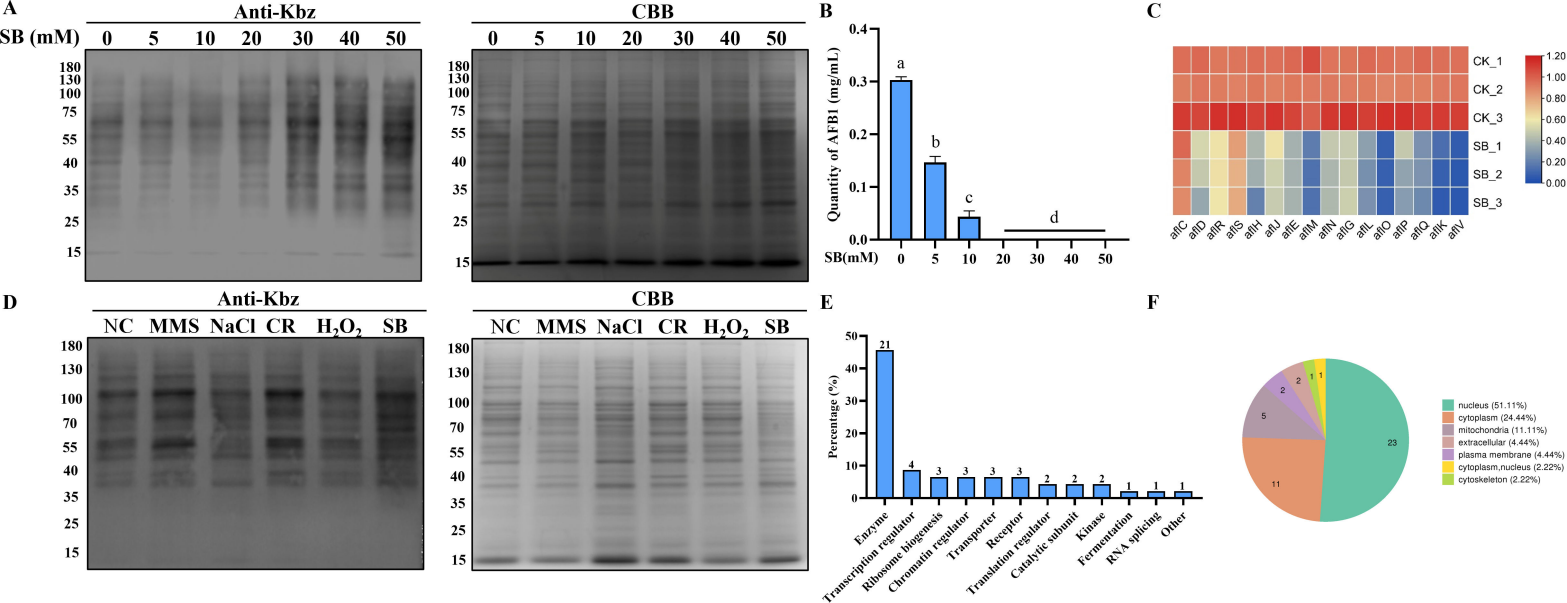
Identification of lysine benzoylation in *A. flavus*. (**A**) Western blotting analysis of benzoylated proteins in response to sodium benzoate (SB) with the indicated concentration in *A. flavus*. Anti-Kbz, western blot with pan anti-benzoyllysine antibody. CBB, Coomassie brilliant blue staining. (**B**) Aflatoxin B_1_ production of *A. flavus* with sodium benzoate treatment at different concentrations. (**C**) Relative expression of aflatoxin synthesis-related genes under 30 mM sodium benzoate (*n* = 3). The little letters above the columns show significant differences (*P* < 0.05). (**D**) Profile of lysine benzoylation in *A. flavus* under various stress conditions. Total proteins were extracted from the cells cultured under 0.02% methyl methanesulfonate (MMS), 1.2 M sodium chloride (NaCl), 500 µg/mL Congo red (CR), 3.5 mM hydrogen peroxide (H_2_O_2_), 30 mM sodium benzoate (SB), or normal condition (NC). (**E**) Classification analysis of benzoylated proteins. (**F**) Subcellular localization of benzoylated proteins.

It was reported that sodium benzoate (SB) could stimulate Kbz by generating benzoyl-CoA (2). To explore the effect of SB in *A. flavus*, we performed WB to detect the Kbz level when exposed to sodium benzoate. Western blot analysis showed a dose-dependent increase of Kbz levels in global proteins after treatment by sodium benzoate (Fig. 1A), but acetylation (Kac) level was decreased (Fig. S1C and D). To gain further insights into the role of benzoylation in *A. flavus*, we quantified the change of growth, sclerotia formation, and aflatoxin production after sodium benzoate stimulated. The results showed a dose-dependent decrease of growth rate, conidiation, sclerotium, and aflatoxin production after sodium benzoate treatment (Fig. 1B and C; Fig. S1E through I). Although the mechanism was unclear, these results implied that lysine benzoylation was likely linked to growth, conidiation, sclerotia formation, and aflatoxin production, which might be an important mechanism for regulating the morphogenesis and secondary metabolism of *A. flavus*.

### Identification of lysine benzoylated proteins and sites in *A. flavus*

To reveal global benzoylation shifts in response to environmental changes, we first assessed benzoylation in *A. flavus* exposed to different stress conditions. The results showed that benzoylation profiles were changed under different stress conditions (Fig. 1D), and overall benzoylation levels were higher in genotoxic stress induced by methyl methanesulfonate, cell wall stress induced by congo red, and sodium benzoate stress. These stresses not only induced change of benzoylation levels, but also affected phenotype and acetylation in *A. flavus* (Fig. S2). These results suggested that Kbz was implicated in the regulation of environmental stress responses.

Previous phenotypic assay and WB data indicated that benzoylation was considerable for fungal development, aflatoxin biosynthesis, and stress response in *A. flavus*. To better characterize the regulatory mechanism of benzoylation, we performed a systematic benzoylome to identify benzoylated proteins and sites, by combining the affinity enrichment and proteomics techniques using the proteins isolated from mycelia under different stress (Fig. S3A). In total, we identified 46 unique benzoylated peptides encompassing 60 benzoylated sites (Table S1), and then all the raw data of MS/MS were uploaded to the China National Gene Bank database (CNP0003875). The distribution of peptide length in proteomic analysis showed that most peptides were between 7 and 15 amino acids in length (Fig. S3B), which is consistent with the properties of trypsin peptides. At the same time, the peptide mass error distribution was close to zero (Fig. S3C), proving the highly accurate and reliable MS data. Of the identified proteins, 34 (74%) proteins contained only one benzoylated site, whereas 12 (26%) proteins had multiple benzoylated sites (Fig. S3D). To determine whether there are common sequence motifs in Kbz peptides, we compared the 10 residues surrounding benzoylated site in the benzoylome of *A. flavus* (Fig. S3E). We identified a preference for polar residue (glutamine) at the +4 positions, whereas lysine was frequently present at the +3, +6, and +9 positions. The different preferences for specific amino acid residues surrounding the benzoylated lysine sites suggested unique substrate preferences in *A. flavus*.

### Functional annotation of benzoylated proteins

To better understand the potential function of benzoylated proteins in *A. flavus*, gene ontology (GO) and Kyoto Encyclopedia of Genes and Genomes (KEGG) annotation analysis were performed, and 46 benzoylated proteins were annotated and classified (Fig. S4A through E). The classification of benzoylated proteins revealed that most of the benzoylated proteins were enzymes (45.7%); however, transcription regulator, ribosome biogenesis, chromatin regulator, transporter, and receptor, along with other types of proteins were also found to be benzoylated, thereby providing additional evidence that Kbz exists among different protein types and could participate in several biological processes (Fig. 1E; Table S1). GO molecular function analysis showed that most benzoylated proteins were associated with organic cyclic compound binding and ion binding (Fig. S4A). Biological process analysis demonstrated that benzoylated proteins were closely associated with various metabolic processes, including primary metabolic process, organic substance metabolic process, and cellular metabolic process (Fig. S4A). In the classification of cellular components, benzoylated proteins were mostly distributed in the organelle, cytoplasm, and membrane-enclosed lumen (Fig. S4A). Enrichment analysis for cellular components demonstrated that benzoylated proteins were mainly distributed in the nuclear lumen and nuclear heterochromatin (Fig. S4B through D). Additionally, KEGG pathway analysis found that metabolic pathways were especially prominent in benzoylated proteins, followed by translation-associated pathways (Fig. S4E). Meanwhile, cell cycle, MAPK signaling pathway, and translation-associated pathway were also found. These results implied that benzoylation could participate in the regulation of various biological processes in *A. flavus*.

Moreover, Wolfpsort software was used to analyze the subcellular locations of benzoylated proteins, and the results showed that most proteins were predicted to be located in the nucleus (51.1%), cytoplasm (24.44%), and mitochondrion (11.11%) (Fig. 1F). This diversity of the subcellular localizations highlights that those various organelles proteins were also modified by Kbz, which was in agreement with the annotation analysis. Altogether, these findings suggested that the benzoylated proteins had a wide distribution of functions and were associated with the translation-associated events and various metabolisms.

### Proteins co-modified by acetylation, succinylation, and benzoylation

Much evidence proved that some PTMs share the same modified proteins and sites, and incorporate crosstalk and substitutions between different PTMs, which leads to fluctuating functional consequences (20). Compared with acetylome and succinylome in our earlier studies, we found less benzoylated proteins and sites than either Kac or Ksucc form in *A. flavus* (Fig. S5A through C). No protein was co-modified with benzoylation and succinylation, and only three proteins were modified by both lysine benzoylation and acetylation, including peroxisomal protein (FoxA), phospholipase D (PLD) and cytochrome P450 (P450) (Fig. S5D). The quantitative analysis of the modified sites found that benzoylated site was completely different from acetylation and succinylation, revealing that benzoylation has itself specific way to form lysine modification, which is different from acetylation and succinylation.

### Identification of a novel Kbz protein AdhB and its benzoylated site K321 in *A. flavus*

**Fig 2:**
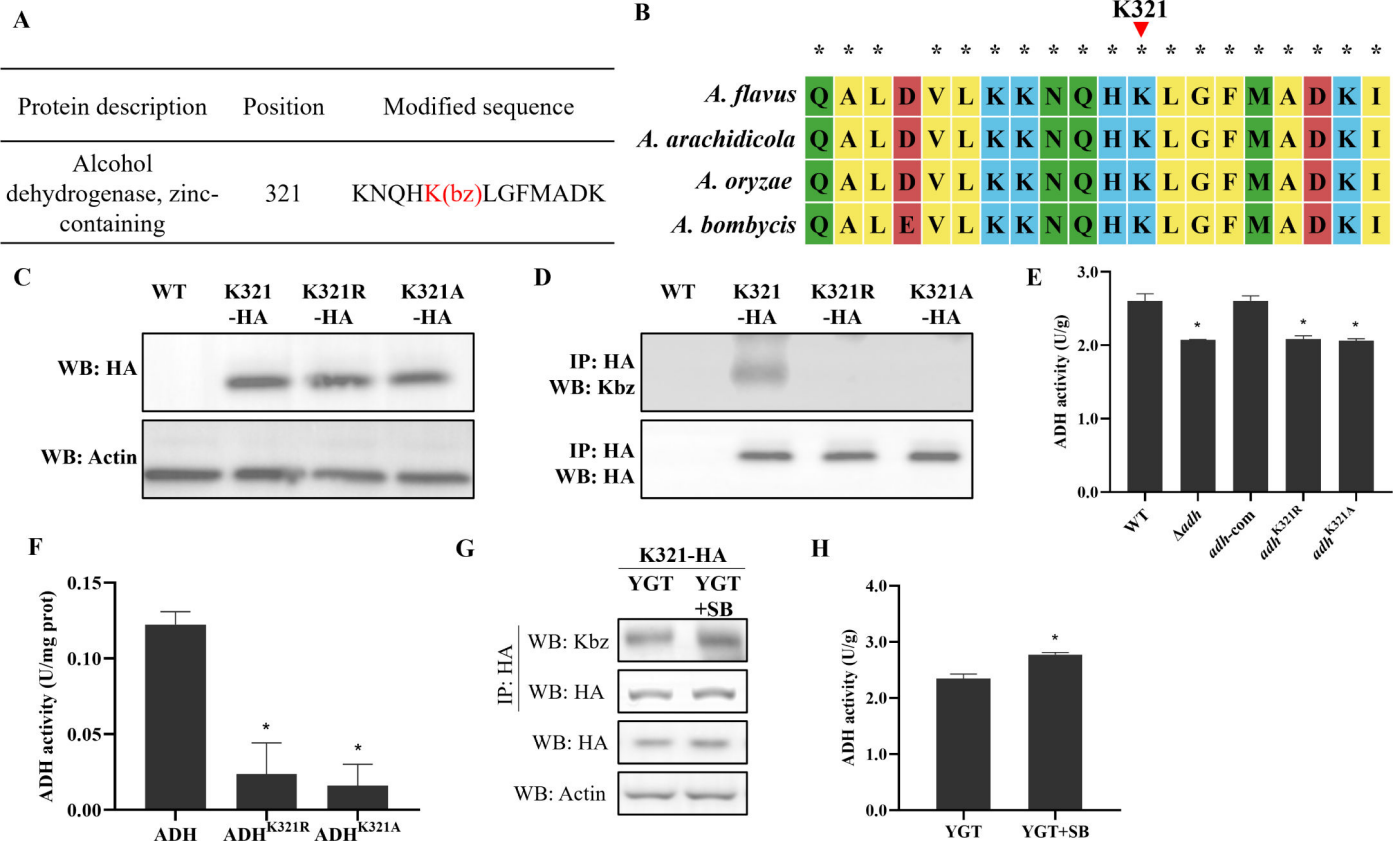
AdhB is a benzoylated protein. (**A**) Tandem mass spectrometry result of lysine benzoylation (Kbz) site for AdhB. (**B**) Conserved site analysis of AdhB. (**C**) Western blot analysis of actin and HA in WT, K321-HA, K321R-HA, and K321A-HA strains. (**D**) Verification of the benzoylated site in AdhB using immunoprecipitation and Western blotting. (**E**) Enzymatic assay of Adh in WT, Δ*adhB, adhB*-com, *adhB*^K321R^, and *adhB*^K321A^ strains. (**F**) Enzymatic assay of AdhB, AdhB^K321R^, and AdhB^K321A^ purified proteins. (**G**) Western blot analysis of benzoylated level and expression level of AdhB protein under SB treatment. (**H**) Enzymatic assay of AdhB under SB treatment.

Alcohol dehydrogenase (ADH) was a glycolytic core enzyme, which was crucial in the regulation of central carbon and energy metabolism. In filamentous fungal pathogens, ADH mediates fungal development and pathogenicity (21). The benzoylomic results in this study identified one benzoylated site on AdhB, located at K321 (Fig. 2A; Fig. S6). Sequence alignment analysis demonstrated that K321, the benzoylated lysine residue, was evolutionarily conserved among different *Aspergillus* species (Fig. 2B). To validate benzoylomic sequencing data of AdhB, K321-HA, K321R-HA, and K321A-HA fusion mutants were generated (Fig. 2C; Fig. S7A), in which arginine (R) and alanine (A) substitutions were used to mimic unacylated and null mutant forms of lysine (K), respectively. The WB results showed that K321 site mutation does not impact AdhB expression (Fig. 2C; Fig. S7B). Then, immunoprecipitation (IP) was performed by using HA antibody, and the IP product was analyzed by immunoblotting with pan-Kbz antibody. The results showed that K321-HA strain exhibited Kbz signal (Fig. 2D), but no Kac and Ksucc signals (Fig. S8), indicating that AdhB was benzoylated protein in *A. flavus*. However, Kbz signal was nearly undetectable in K321R-HA and K321A-HA strains (Fig. 2D), confirming that K321 of AdhB was indeed benzoylated site in *A. flavus*.

### Mutation of benzoylated site K321 inhibits AdhB activity in *A. flavus*

ADH was a crucial enzyme in short-chain fatty acid metabolism, which could catalyze the reversible conversion of ethanol to acetaldehyde (22). To evaluate the effect of benzoylation on the AdhB, we compared the alcohol dehydrogenase activity among the WT, *adhB* deletion mutant (Δ*adhB*), complementation mutant (*adhB*-com), and point-mutants (*adhB*^K321R^ and *adhB*^K321A^) (Fig. S7C through E). Benzoylated points (K/R and K/A) and *adhB* deletion mutants showed reduced alcohol dehydrogenase activity compared to WT and complementation strains (Fig. 2E). Meanwhile, mutations in K321 site also led to a significant decrease in the enzymatic activity of purified AdhB protein *in vitro* (Fig. 2F). The DSC results showed that the thermal stability of AdhB^K321R^ and AdhB^K321A^ proteins was lower than AdhB protein (Fig. S9). These results suggested that K321 was an important regulatory benzoylation site that controls AdhB activity.

To further clarify the effect of benzoylation on AdhB activity, we determined the benzoylation level and enzyme activity of AdhB protein after sodium benzoate (20 mM) treatment. The results showed that addition of sodium benzoate can cause a significant increase in benzoylation level of AdhB and intracellular alcohol dehydrogenase activity (Fig. 2G and H), but did not affect the AdhB expression, revealing that sodium benzoate can enhance its enzymatic activity by inducing benzoylation levels.

### Mutation of benzoylated site in AdhB caused increased aflatoxin production

**Fig 3:**
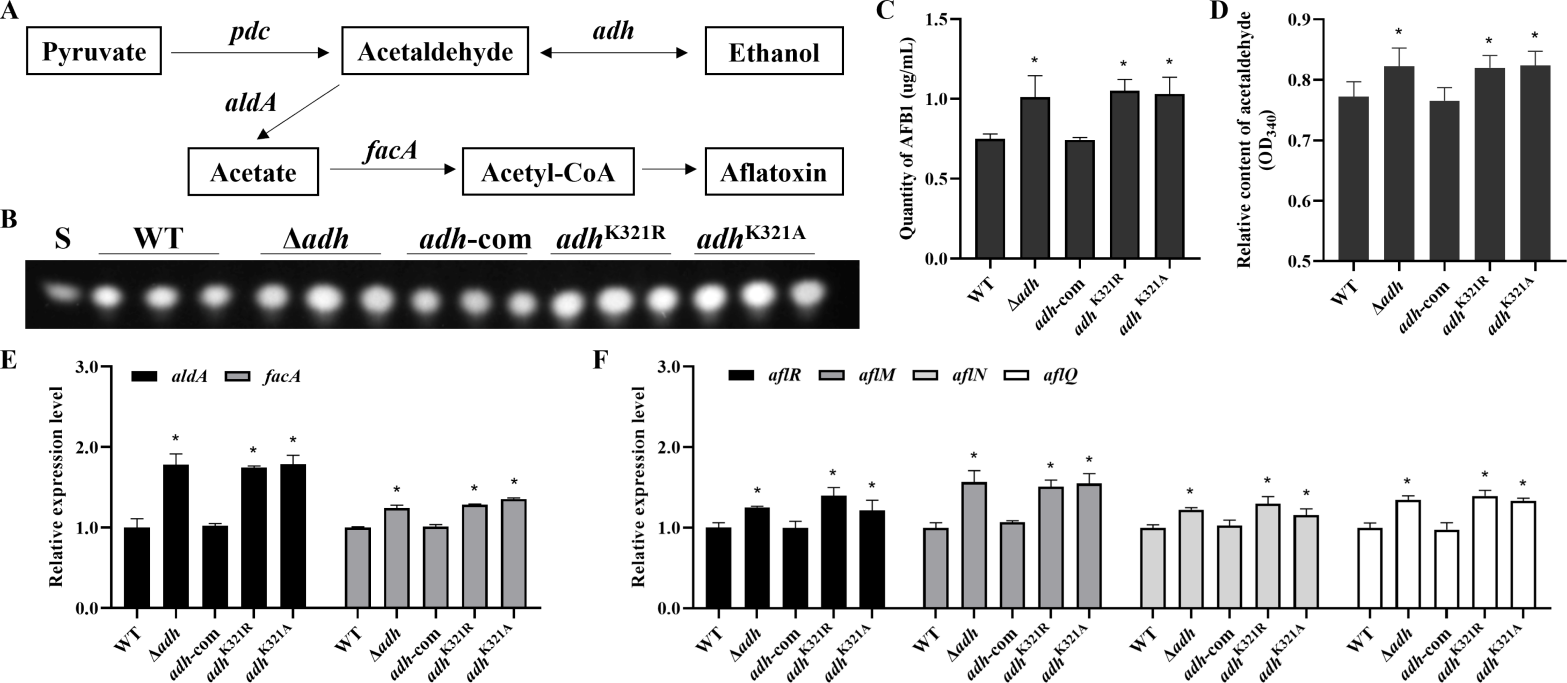
Benzoylation site of AdhB exerts an important role in alcohol dehydrogenase activity and aflatoxin biosynthesis. (**A**) Schematic depiction of Adh function in *A. flavus*. (**B**) TLC assay of AFB_1_ production by the WT and *adhB* mutants cultured in YES liquid media at 29°C for 6 days. S indicates AFB_1_ standard. (**C**) Quantification analysis of AFB_1_ in TLC results by optical density (*n* = 3). (**D**) Relative content of acetaldehyde in WT and *adhB* mutants (*n* = 3). (**E**) Relative transcript levels of *aldA* and *facA* in different strains (*n* = 3). (**F**) Relative transcript levels of aflatoxin biosynthesis-associated genes (*aflR*, *aflM*, *aflN*, and *aflQ*) in different strains (*n* = 3). Asterisks represent statistically significant differences (*P*  <  0.05).

Aflatoxin was a critical virulence factor in *A. flavus*, and its biosynthetic precursor acetyl-CoA could be provided by acetate generated from β-oxidation and/or acetaldehyde (23), and the acetaldehyde formation was regulated by ADH (Fig. 3A), which implied that Kbz may regulate the aflatoxin biosynthesis by affecting the activity of AdhB in *A. flavus*. To validate this conjecture, we quantified the aflatoxin B_1_ production in WT and above *adhB* mutants. Compared with WT and complementation strains, all *adhB* mutants produced more aflatoxin B_1_ (Fig. 3B and C). To further identify the regulatory mechanisms underlying the role of AdhB in aflatoxin production, we detected the acetaldehyde production. Compared to WT and complementation strains, *adhB* deletion and point mutants exhibited higher acetaldehyde production (Fig. 3D). Then, we examined the expression level of acetyl-CoA formation-related genes (acetaldehyde dehydrogenase *aldA* and acetyl-CoA synthetase *facA*) and aflatoxin biosynthesis cluster genes (*aflR*, *aflM*, *aflN*, and *aflQ*). The qPCR data showed that the transcript levels of abovementioned genes were all increased in the *adhB* deletion and point mutants, compared with those in the WT and complementation strains (Fig. 3E and F), suggesting that AdhB may impact the aflatoxin production through the regulation of acetyl-CoA formation. Altogether, AdhB and its benzoylation sites were involved in regulating aflatoxin biosynthesis in *A. flavus*.

### AdhB regulates fungal development

To systematically evaluate the biological functions of *adhB* in *A. flavus*, we analyzed the transcriptional profiles of *adhB* during vegetative growth (VG), conidiation (CON), sclerotia development (SCR), and aflatoxin synthesis (AFS). The expression patterns revealed that *adhB* was highly expressed in VG stage, followed by the CON and AFS stages, and finally by SCR stage, suggesting that *adhB* not only plays an important role in aflatoxin synthesis, but also may in development of *A. flavus* (Fig. S10A).

To investigate the potential roles of *adhB* and its K321 site in hyphal growth and conidiation formation in *A. flavus*, WT and all mutants were incubated in YGT media and glycerol media (1% glycerol as the sole carbon source) at 37°C for 5 days. The results showed that *adhB* deletion and benzoylated point mutants displayed a defect in development with fewer conidia and less growth rate (Fig. S10B through F). Next, we examined the expression level of *abaA* and *brlA* that was essential for conidiation formation, and data showed that *abaA* and *brlA* were significantly decreased in Δ*adhB*, *adhB*^K321R^, and *adhB*^K321A^ (Fig. S10G). These results indicated that *adhB* and its K321 site were important for asexual spore production.

Sclerotia not only acts as survival structure to adapt adverse environmental conditions, but also was prerequisite for sexual reproduction (24). The statistical results showed that *adhB* deletion and benzoylated point mutants produced abundant sclerotia, compared to WT and *adhB*-com strains (Fig. S11A and B). Consistent with this, the transcription levels of key transcription factors for sclerotia formation (*nsdC* and *sclR*) were both upregulated significantly in the *adhB* deletion and benzoylated point mutants (Fig. S11C). Overall, benzoylation site K321 could modulate fungal development by regulating the AdhB activity in *A. flavus*.

### AdhB and its benzoylated site K321 are important for fungal pathogenicity

**Fig 4:**
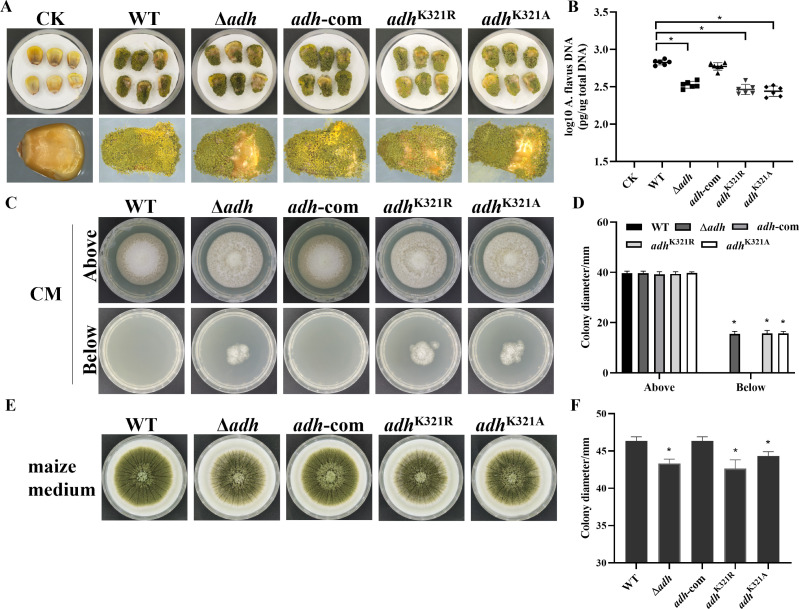
K321 of AdhB was important for pathogenicity. (**A**) Phenotypic observation of WT, Δ*adhB, adhB*-com, *adhB*^K321R^, and *adhB*^K321A^ strains in maize kernels. (**B**) Maize seeds were harvested, and fungal burdens were estimated by quantification of fungal DNA using qPCR (*n* = 6). (**C**) WT and mutants were grown on CM media overlaid with a cellophane layer for 4 days (above), and the plates were further incubated for 1 day after removing the cellophane layer (below). (**D**) Statistical analysis of colony diameter in CM media (*n* = 3). (**E**) Morphology of WT and *adhB* mutants in maize medium. (**F**) Statistics analysis of colony diameter in WT and *adhB* mutants (*n* = 3). Asterisks represent statistically significant differences (*P*  <  0.05).

To determine the effects of AdhB and its benzoylation site on pathogenicity, live maize seeds were inoculated with spores from the WT, Δ*adhB*, *adhB*-com, *adhB*^K321R^, and *adhB*^K321A^, then fungal burden, conidia, and aflatoxin production were examined. Meanwhile, inoculated with sterile water as controls (CK). We found that *adhB* deletion and benzoylated point mutants significantly reduced the fungal DNA burden and conidia production, but increased AFB_1_ production (Fig. 4A and B; Fig. S12). These results indicated that *adhB* and its K321 site contribute to seed colonization of *A. flavus* on host maize seed.

During infection, fungal hyphae penetrate the host tissue, and then obtain host nutrients for proliferation. To further clarify the effect of *adhB* and its benzoylation site in invasion process, we examined the fungal penetration. All strains were cultured on medium (CM and YGT) covered with cellophane, and the colony diameter was measured after removal of cellophane. The results showed that *adhB* deletion and benzoylated point mutants exhibited higher penetration (Fig. 4C and D; Fig. S13A and B). Considering that lipase was involved in the fungal penetration (25, 26), we analyzed the lipase activity by measuring the inhibition rate of mutants to glyceryl tributyrate. The results showed that the relative inhibition rate of glyceryl tributyrate to the colony growth of Δ*adhB*, *adhB*^K321R^, and *adhB*^K321A^ were significantly higher than that of WT and complementation strains (Fig. S13C and D), hinting that the *adhB* deletion and K321 mutation might dramatically increase the lipase activity in *A. flavus*. Since a conflict between higher penetration and reduced fungal burden was observed, we hypothesized that the mutation of *adhB* might have caused the inhibited vegetative growth on maize, so we further detected the vegetative growth of WT and *adhB* mutants on maize medium. Our results showed that all *adhB* mutants exhibited lower growth rates than WT and complementation strains (Fig. 4E and F), which provides a proof for our conjecture. Despite the greater penetration and lipase activity of the *adhB* mutants, a weak growth was detected in maize medium and maize, ultimately leading to a reduced fungal burden in maize kernels. All these findings suggested that AdhB and its benzoylation site K321 were important factors for fungal pathogenicity in *A. flavus*.

### K321 site of AdhB is required for maintaining cell wall integrity

**Fig 5:**
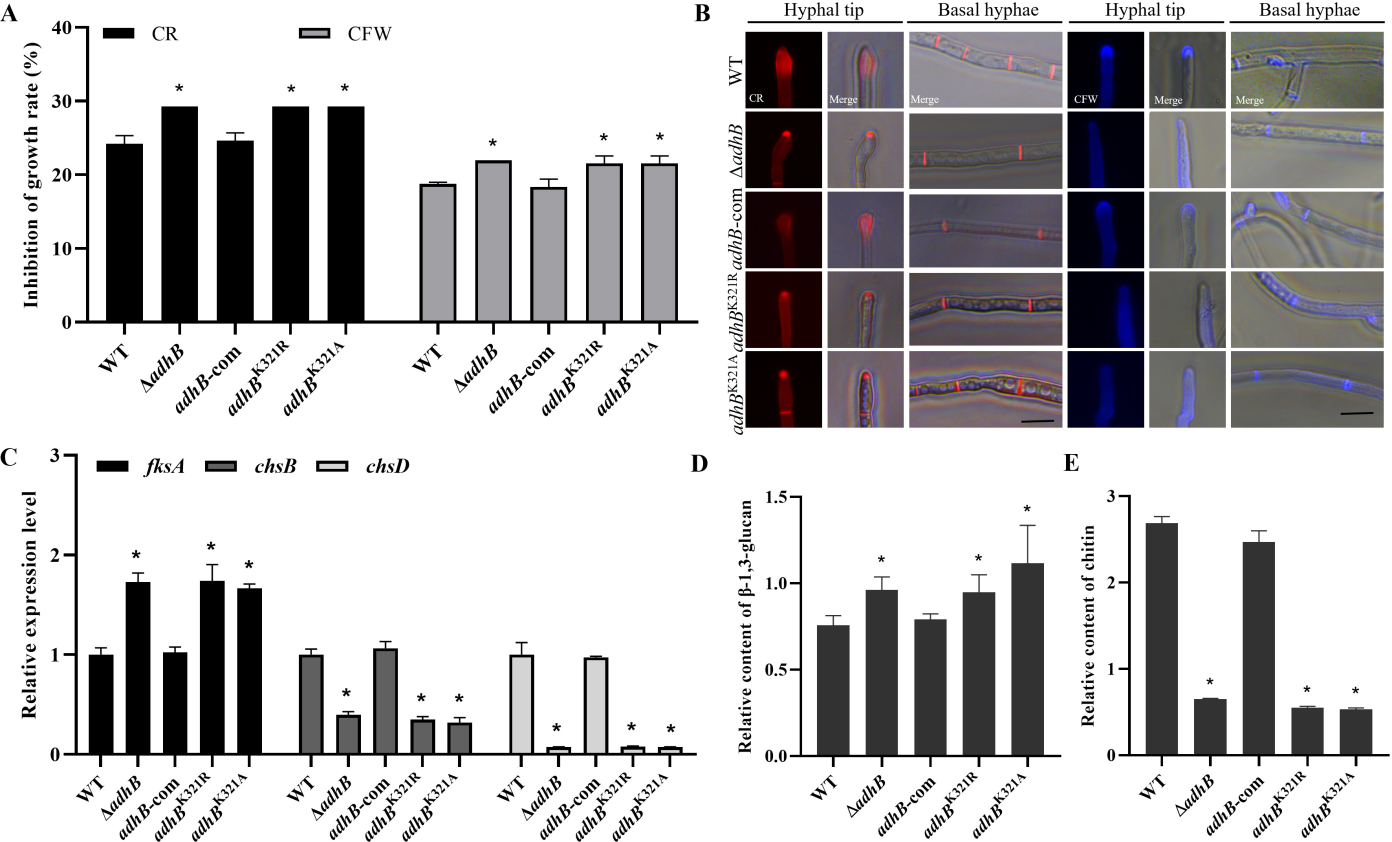
K321 site of AdhB is involved in cell wall stress response. (**A**) Statistical analysis of inhibition rate by CR and CFW (*n* = 3). (**B**) CR and CFW staining analysis of hyphal tip and basal hypha in different mutants. WT, Δ*adhB*, *adhB*-com, *adhB*^K321R^, and *adhB*^K321A^ mutants were inoculated into YGT liquid media and grown overnight, and mycelium was collected and stained with 10 mg/mL CR (left) or CFW (right) for 5 min, then observed by fluorescent microscopy. Bar = 10 µm. (**C**) Transcriptional expression levels of *fksA*, *chsB*, and *chsD* genes (*n* = 3). (**D**) Relative content of β-1,3-glucan (*n* = 3). (**E**) Relative content of chitin (*n* = 3). Asterisks represent statistically significant differences (*P*  <  0.05).

The fungus cell wall is the first line of defense against environmental stress (27). To characterize the roles of *adhB* in response to cell wall stress, the sensitivity of Δ*adhB* was tested to cell wall damaging stress (62.5–500 μg/mL CR and 50–400 μg/mL CFW), and the results showed that the lack of *adhB* increases the sensitivity to the cell wall perturbing agents in all tested concentration (Fig. S14A through C). To further figure out the Kbz role in cell wall stress response, WT and *adhB* mutants were inoculated onto YGT medium with 500 µg/mL CR or 400 µg/mL CFW. The inhibition rate of *adhB* deletion and benzoylated point mutants were remarkedly greater than those of WT and complementation strains (Fig. 5A; Fig. S14D). Because glucan and chitin were the major components of fungal cell wall, CR, and CFW could bind to β-1, 3-glucan, and chitin, respectively (28). So, we checked for possible deposition in WT and *adhB* mutants through CR and CFW staining. Microscopic examination indicated that the *adhB* deletion and benzoylated point mutants displayed abnormal cell wall structure, which had excessive glucan deposition and reduced chitin deposition at hyphal tips (Fig. 5B). Therefore, we speculate that AdhB leads to cell wall deposition mainly by interfering with the normal expression of β-1, 3-glucan and chitin. To further verify this, we not only examined the transcription levels of β-1,3-glucan synthase gene (*fksA*) and chitin synthase genes (*chsB* and *chsD*), but also quantitated the β-1,3-glucan and chitin content. The qPCR data showed that the expression of *fksA* was upregulated in the *adhB* deletion and benzoylated point mutants, while the expression of *chsB* and *chsD* was downregulated (Fig. 5C). The quantitation results also revealed that *adhB* deletion and benzoylated point mutants exhibited increased β-1,3-glucan content and decreased chitin content (Fig. 5D and E), which was consistent with our speculation. Totally, above results illustrated that the defective benzoylation of AdhB can trigger abnormal cell wall deposition by affecting the distribution and synthesis of glucan and chitin, which also demonstrated the important role of AdhB and its benzoylation site K321 in maintaining cell wall integrity.

### GcnE has benzoyltransferase activity *in vivo* and *in vitro*

**Fig 6:**
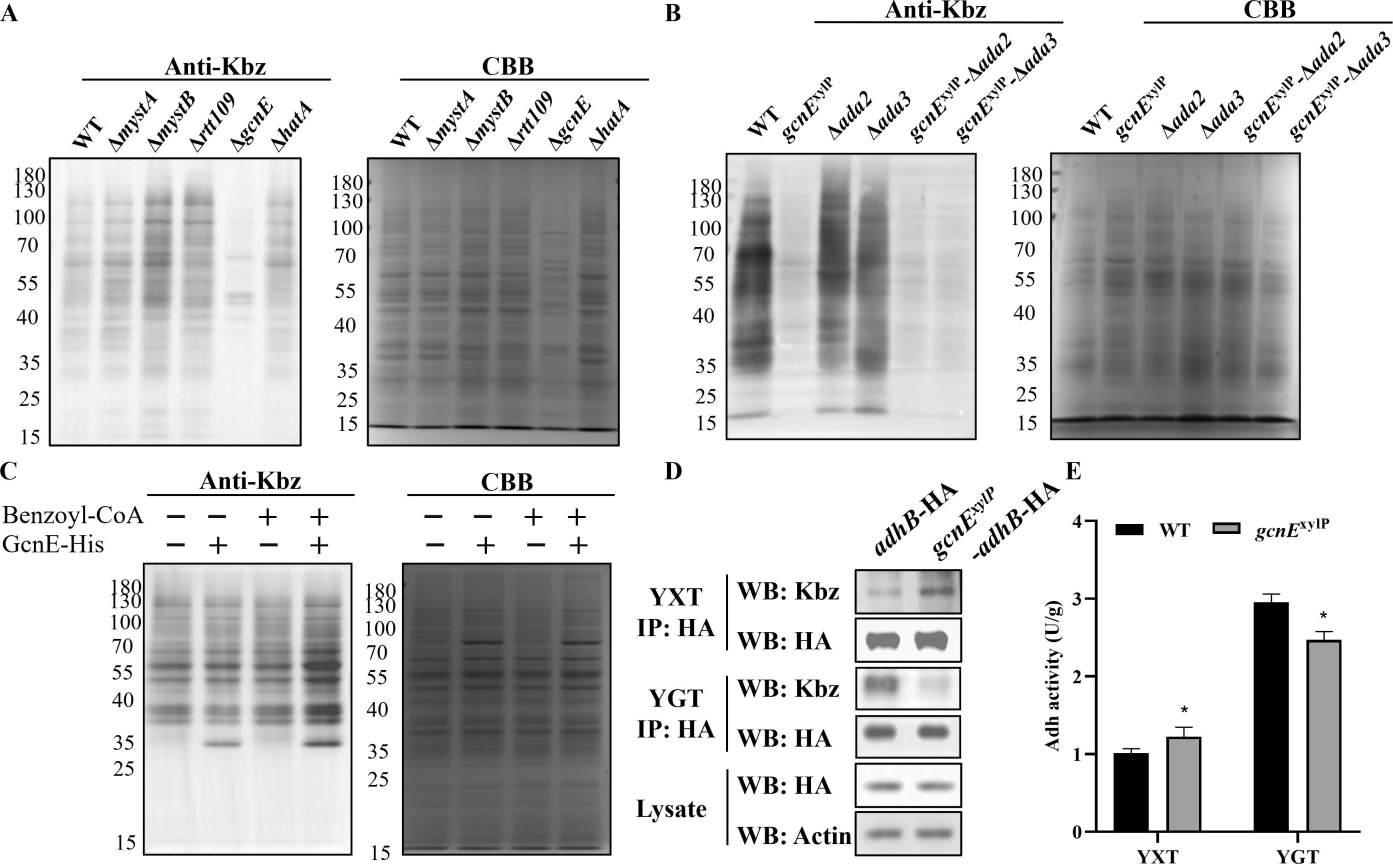
GcnE has benzoyltransferase activity *in vitro* and *in vivo*. (**A**) Western blot analysis of Kbz in WT, Δ*mystA*, Δ*mystB*, Δ*rtt109*, Δ*gcnE*, and Δ*hatA* strains. (**B**) Western blot analysis of Kbz in WT and *gcnE*^xylP^, Δ*ada2,* Δ*ada3*, *gcnE*^xylP^-Δ*ada2*, and *gcnE*^xylP^-Δ*ada3* strains. (**C**) *In vitro* lysine Kbz activity of GcnE protein by western blotting assays. (**D**) Densitometric quantification of the *in vivo* AdhB benzoylation and the expression levels in *adhB*-HA and *gcnE*^xylP^-*adhB*-HA strains under YGT or YXT condition. (**E**) The Adh enzymatic activity of WT and gcnE^xylP^ strains under YGT or YXT condition.

Benzoylation is a dynamic process controlled by debenzoylase and benzoyltransferase (2, 29); however, no work has been reported for benzoyltransferase in pathogenic fungi. To search for potential lysine acyltransferases (KATs) responsible for AdhB benzoylation in *A. flavus*, we performed WB assays to examine Kbz levels of non-essential acyltransferase deletion mutants *in vivo*. Considering some acyltransferase mutants have been constructed in our laboratory, including Δ*rtt109* (30), Δ*gcnE* (31), Δ*mystA*, and Δ*mystB* (32)*,* we construct another acyltransferase *hatA* deletion (Δ*hatA*) strain in this study (Fig. S15A and B). Consistent with expectation, acyltransferases deletion mutants exhibited lower acetylation levels than WT (Fig. S16A and B). Moreover, the level of Kbz was only significantly downregulated in the *gcnE* deletion mutant, but not in other acyltransferase deletion mutants (Fig. 6A; Fig. S16B). Considering that *gcnE* deletion has a significant defect on fungal growth and total protein content under the same culture conditions (31), we further constructed *gcnE*^xylP^ mutant, with insertion of screening marker *pyrG* and xylose inducible promoter upstream of *gcnE* (Fig. S15C and D). Expression from the xylP promoter was repressed with glucose, induced by xylose, and super-induced by xylan. Compared to WT, Kbz of *gcnE*^xylP^ mutant was increased under the xylP inductive condition (2% xylose or xylan), but decreased under xylP repressive condition (2% glucose) (Fig. S17). The above results indicated that *gcnE* has benzoyltransferase activity in *A. flavus*. GcnE is the major member of ADA complex, and the role of ADA complex in Kbz is unclear, so we further constructed single deletion (Δ*ada2* and Δ*ada3*) and double deletion (*gcnE*^xylP^-Δ*ada2* and *gcnE*^xylP^-Δ*ada3*) mutants of the complex (Fig. S18A). The Kbz and Kac level was significantly decreased in all *gcnE*-related mutants (*gcnE*^xylP^, *gcnE*^xylP^-Δ*ada2*, and *gcnE*^xylP^-Δ*ada3*), but not in other single deletion mutants (Δ*ada2* and Δ*ada3*) of the complex member (Fig. 6B; Fig. S16C and D), suggesting that GcnE catalyzes benzoylation and acetylation modification in an ADA complex-independent manner.

To examine whether GcnE has benzoyltransferase activity *in vitro*, we expressed AflGcnE in *Escherichia coli* and obtained the purified AflGcnE proteins (Fig. S19A). *In vitro* enzymatic activity experiments showed that Kbz signal was increased in some protein bands with GcnE-His and benzoyl-CoA treatment, but it did not happen with only benzoyl-CoA treatment (Fig. 6C), which suggests that GcnE functions as a transfer-modification enzyme to add benzoylation *in vitro*.

### GcnE is involved in K321 benzoylation of AdhB and enzymatic activity

To test whether GcnE can mediate the benzoylation of AdhB, we constructed *gcnE*^xylP^-*adhB*-HA (Fig. S18A and B) and examined the Kbz and expression of AdhB in *gcnE*^xylP^-*adhB*-HA and *adhB*-HA (K321-HA) mutants. As expected, induction of GcnE increased the K321 benzoylation level of endogenous AdhB, and repression of GcnE decreased the K321 benzoylation level of AdhB, but did not affect the expression of AdhB (Fig. 6D). Hence, we speculated that GcnE could directly regulate the endogenous AdhB activity. To validate our conjecture, we determined the activity of endogenous Adh in WT and *gcnE*^xylP^ strains. The results showed that induction of GcnE significantly enhanced the Adh activity, whereas inhibition of GcnE led to a decrease in Adh activity *in vivo* (Fig. 6E). These results further supported that GcnE was the benzoyltransferase of AdhB in *A. flavus*, and GcnE modulates Adh activity by regulating benzoylation of AdhB.

### Both BRO and GNAT domains are important in benzoyltransferase activity of GcnE

GcnE consists of the catalytic domain (GNAT) and acylation lysine residues binding domain (Bromodomain, BRO). To elucidate the function of different domains, the domain deletion mutants *gcnE*^ΔGNAT^ and *gcnE*^ΔBRO^ were constructed (Fig. S18C and D), and our qPCR data showed that the expression level of *gcnE* gene in *gcnE*^ΔBRO^ and *gcnE*^ΔGNAT^ were increased (Fig. S18F). The western blotting results showed that Kbz and Kac were decreased in all mutants, with the greatest decrease in the *gcnE*^xylP^ mutant, followed by *gcnE*^ΔGNAT^, then by *gcnE*^ΔBRO^ (Fig. S20A through C), and the Adh activity was decreased in *gcnE*^xylP^, *gcnE*^ΔBRO^, and *gcnE*^ΔGNAT^ mutants (Fig. S20D), revealing that BRO and GNAT domains have important roles in benzoyltransferase and acetyltransferase activity of GcnE.

GcnE has been proven to play important biological functions in *A. flavus* (31), but the function of domains was unclear, so we examined the role of BRO and GNAT domains in fungal growth, conidiation, sclerotia formation, aflatoxin production, and stress response of *A. flavus*. Phenotypic assays showed that *gcnE*^ΔGNAT^ mutants exhibited similar phenotypes to the *gcnE*^xylP^ mutant, including failing to produce conidia, sclerotia, and AFB_1_, and showing more sensitivity to cell wall stress and DNA damage stress (Fig. S20E through J and S21). Meanwhile, deletion of BRO domain also results in reduced conidia, sclerotia, and AFB_1_ production, as well as greater sensitivity to DNA damage stress (Fig. S20E through J and S21). Taken together, these data demonstrated that the GNAT and BRO domains were crucial for fungal development and aflatoxin biosynthesis in *A. flavus*.

### Benzoyltransferase activity depended on catalytic residue E139 in GcnE

Previous studies indicated that E139 was a conserved and important catalytic site in GcnE (33). To investigate the function of E139 in GcnE, we successfully generated point mutation strain *gcnE*^E139H^ (Fig. S18E), and qPCR data showed that the expression level of *gcnE* gene in *gcnE*^E139H^ was increased (Fig. S18F). The structure simulation results showed that E139 was also located in histone substrate binding cleft (Fig. S19B), which is similar to homologous site E173 in yeast (34). The Western blotting results showed that Kbz and Kac were also significantly decreased in *gcnE*^E139H^ mutant (Fig. S20B and C); meanwhile, the enzymatic data showed that Adh activity was decreased in *gcnE*^E139H^ strain (Fig. S20D), suggesting that E139H site was important for benzoyltransferase and acetyltransferase activity of GcnE.

As expected, *gcnE*^E139H^ mutant displayed *gcnE*^ΔGNAT^ and *gcnE*^xylP^ mutant-like phenotypes in growth, conidiation, sclerotia formation, aflatoxin production, and stress response. In detail, when glutamate 139 in GcnE protein was substituted by histidine, fungal conidiation, sclerotia formation, and AFB_1_ production were almost completely suppressed, and exhibited more sensitivity to cell wall stress and DNA damage stress (Fig. S20E through J and S21). All these results revealed that the conserved E139 site was important for biological functions of GcnE.

## DISCUSSION

Mounting evidence indicated lysine acylations were associated with cellular physiology and pathogenesis in eukaryotes (15, 35). Lysine benzoylation has been identified as a new type of PTM which associated with active transcription. Although it has been demonstrated that benzoylation has multiple substrates including histone benzoylated sites and non-histone proteins, non-histone benzoylated sites were only identified in yeast (8), but the effect of Kbz on modified protein is still unknown. In this study, we first proved that lysine benzoylation was widely distributed among prokaryotes and eukaryotes. Further, we identified 60 benzoylated sites on 46 proteins through proteome-wide screening in *A. flavus*. Among these, we demonstrated that K321 of AdhB protein was an important benzoylated site, and point mutation of benzoylated site of AdhB leads to decreased enzymatic activity and further affects the fungal morphology development, aflatoxin biosynthesis, pathogenicity, and stress response. Moreover, we found that GcnE was a benzoyltransferase for AdhB. Totally, benzoyltransferase GcnE mediates the benzoylation of AdhB protein, then regulates its enzymatic activity, which in turn affects the pathogenicity of *A. flavus*.

Benzoate is an essential precursor for benzoylation, and exogenous addition of sodium benzoate could lead to an increase of benzoyl-CoA content in cells and further induce histone benzoylation (2, 8). In this study, we found that sodium benzoate treatment significantly enhanced the Kbz level on the total protein of *A. flavus* in a dose-dependent manner, and also significantly inhibited the fungal growth, conidiation, sclerotia formation, and aflatoxin production in *A. flavus*. It is similar to previous stud that sodium benzoate acted as a common preservative, can effectively inhibit the growth and aflatoxin production of *A. flavus* (36). Likewise, environmental stresses also lead to Kbz changes on the total protein of *A. flavus*. These results suggested that benzoylation may have broad functions to regulate the morphogenesis, secondary metabolism and stress response in *A. flavus*. Moreover, lysine benzoylation was recently reported as a histone mark in eukaryotic cells (2). This modification has been studied in yeast, mouse, and human cells (2, 8), but Kbz in fungal pathogens has not been reported till now. In this study, we performed a comprehensive analysis of Kbz in *A. flavus* and found that many proteins involved in amino acid metabolism, such as CBS, BG1, D3PD, Fox2, HisD, and LysF, were identified as putative benzoylated proteins. *LysF* and *fox2* gene deletion led to attenuated virulence in *A. fumigatus* (37) and *C. albicans* (38), respectively. Accordingly, we hypothesize that some proteins involved in energy metabolism that regulate the development and virulence of *A. flavus* might be influenced by benzoylation and would be as potential target for antifungal drugs. Benzoylated proteins are not only mainly annotated into metabolic-related pathways and ribosome biogenesis process in *A. flavus*, but are also involved in ribosome biogenesis, glycolytic process, gluconeogenesis, and rRNA processing in yeast (8). In summary, lysine benzoylation has a wide range of biological functions in eukaryotes.

Mounting evidences suggest that crosstalk between post-translational modifications often occurs (39–41), which enriches the regulatory network of post-translational modifications. This crosstalk can be broadly classified into three categories. The first class is the same enzyme that can regulate multiple post-translational modifications simultaneously, such as Gcn5 has the transferase activity of acetylation, crotonylation, and benzoylation (8, 33), and SIRT2 also has deacylation activity of acetylation, propionylation, butyrylation, crotonylation, benzoylation, and methacryloylation (2, 5, 42–44). The second class is that multiple post-translational modifications were present on the same protein, for example, malate dehydrogenase (MDH) protein was simultaneously modified by succinylation, acetylation, malonylation, crotonylation, and 2-hydroxyisobutyrylation in rice seed (45). And 250 crotonylated sites within 177 crotonylated proteins were also co-modified by acetylation and succinylation in *C. albicans* (46). K6 acetylation of huntingtin exon1 (Httex1) could reverse the inhibitory effect triggered by T3 phosphorylation (47). The last class is that the modified protein plays an important role in another post-translational modification, for instance, E3 ligase Trim24 promotes acetylation for downstream Stat6 by catalyzing ubiquitination at the K199 of acetyltransferase CBP (48). Compared to acetylome and succinylome data in *A. flavus*, only three proteins (PLD, Fox2, and P450) were co-modified by benzoylation and acetylation with different modified lysine sites. Further research is required to investigate the functions and relationships of acetylation and benzoylation on the co-modified proteins. Meanwhile, we found five benzoylated proteins which associated with other post-translational modifications, including ribonucleic acid methyltransferase Nop2, ubiquitin C-terminal hydrolase Uch, histone deacetylase Set3 complex subunit Hos4, and two proteins containing kinase structural domains (B8NR43 and B8NC83). It is reported that Nop2 was able to methylate C2870 and C2278 of 25S rRNA in yeast (49), Uch-L3 could regulate Smad1 ubiquitination and osteoblast differentiation (50), and the Set3 complex was able to participate in the regulation of infectious growth of *Magnaporthe oryzae* (51). Alternatively, our results showed that both sodium benzoate and environmental stress were able to induce the change of Kbz and Kac levels in *A. flavus*. These results implied that benzoylation has potential crosstalk with methylation, ubiquitination, acetylation, and phosphorylation, but further studies are required to prove it.

Among all metabolic pathways, glycolytic enzymes were identified to be putative benzoylated and involved in the conversion of glucose to pyruvate in yeast (8). In *A. flavus*, we found alcohol dehydrogenase B (AdhB) within the glycolysis/gluconeogenesis pathway was benzoylated, indicating a potentially conserved role of benzoylation in regulating the glycolytic pathway. Consistent with acylomic data, we found that AdhB was bona fide benzoylated protein that could bind with pan-Kbz antibody rather than pan-Kac antibody and pan-Ksucc antibody in IP-WB experiment. The ADH was responsible for the reversible oxidation of alcohols to aldehydes (22). By quantifying the alcohol dehydrogenase activity, we found a significantly decreased enzymatic activity in *adhB* deletion mutant, but not completely abolished. It means that AdhB has ethanol dehydrogenase activity and other isozymes may also be present in *A. flavus*. It is consistent that ADH has many family members, for example, there are three ADH members (ADH1, ADH2, and ADH3) in *S. cerevisiae* (22), and two (ADH I and ADH II) in *Aspergillus nidulans* (52). Meanwhile, sodium benzoate increased the enzymatic activity of AdhB by upregulating its benzoylation level, and GcnE repression decreased the enzymatic activity of AdhB by downregulating its benzoylation level, indicating that the benzoylation was crucial for function execution of AdhB. In addition to benzoylation, other acylations could also directly affect the enzymatic activity of the modified substrate. In HEK293T cells, G6PD was negatively regulated by acetylation on K403 with loss of enzymatic activity (53), and succinylated enolase 1 also resulted in reduced enolase enzymatic activity *in vivo* and *in vitro* (54). In *Ustilaginoidea virens*, the 2-hydroxyisobutyrylation-deficient of Uvslt2 leads to defected enzymatic activity (35). Therefore, acylation modification has various effects on modified enzymes.

Acetaldehyde was a key metabolite for aflatoxin production, which was converted to ethanol by alcohol dehydrogenase or to acetate by aldehyde dehydrogenase. Then acetate was converted to acetyl coenzyme by acetyl coenzyme A synthetase (55), and acetyl coenzyme A was the initial substrate of aflatoxins biosynthetic pathway. Previous stud showed that external SOD could inhibit aflatoxin production by upregulating the transcription level of alcohol dehydrogenase in *A. flavus* (55). Therefore, alcohol dehydrogenase might be involved in aflatoxin synthesis in *A. flavus*. Combined with aflatoxin production, acetaldehyde production, and qPCR data, we hypothesize that AdhB may affect acetyl-coenzyme A formation by negatively regulating acetaldehyde synthesis, which leads to a change in aflatoxin production. Similarly, *adh1* deletion mutant produced significantly increased acetaldehyde than WT in *S. cerevisiae* and *Metarhizium acridum* (56, 57).

For successful infection, fungal pathogens have evolved complex regulatory mechanisms to facilitate penetration and colonization (58). In pathogenicity assay, the results showed that Δ*adhB* mutant exhibited greater penetration, higher lipase activity, lower fungal growth rate, and fungal burden compared to WT, revealing that AdhB regulates the pathogenicity of *A. flavus* by affecting the fungal penetration, vegetative growth, and colonization. Similarly, the role of AdhB in pathogenicity has been reported in other fungi. For instance, Adh was required for the pathogenicity of *Botrytis cinerea* and *C. albicans* (21, 59). Deletion of *alcC* (ADH3 orthologs) in *A. fumigatus* alters the pathogenesis of invasive pulmonary Aspergillosis through reduced fungal growth *in vivo* and increased inflammatory response (60). These observations fully illustrated that alcohol dehydrogenase plays an important role in the pathogenicity of fungal pathogens. Moreover, phenotypic assay showed that *adhB* deletion resulted in decreased conidiation and increased sclerotia formation, which indicated that *adh* was important for fungal development. The importance of ADH for development has also been reported in other fungi. Such as Δ*adh* mutant has a defect in growth of *C. albicans* (59). Similarly, Δ*bcadh1* mutant exhibited reduced conidia formation and increased sclerotia number in *B. cinerea* (21). However, in *M. acridum*, the biomass and conidiation of MaADH1 deletion mutant had no significant difference compared with WT, while decreased significantly under low oxygen conditions (57). These observations suggest that ADH regulates morphogenesis and pathogenicity in a fungal species-specific manner.

Cell wall assembly was required for hyphal growth in filamentous fungi (61). Our findings suggested that *adhB* was required for maintaining cell wall integrity in *A. flavus*, and the deletion mutant was more sensitive to CR and CFW treatment. The cell wall stress agents CR and CFW disrupt cell wall integrity by binding β-1,3-glucan and chitin, respectively (62, 63). It has been reported that chitin and glucan were the major components of the cell wall for filamentous fungal, and their normal synthesis and distribution were important to maintain cell wall integrity and polar hyphal tip growth (64). However, our microscopic examination indicated that Δ*adhB* mutant had increased β-1,3-glucan and reduced chitin deposition on the hyphal tips. Chitin polymers were synthesized by chitin synthase (Chs) genes, therein, ChsB plays a key role in hyphal tip growth in filamentous fungi (65), and Chs members (1, 3, and 4) were involved in cell wall synthesis and maintaining cell wall integrity in *Neurospora crassa* (66). Consistent with observed abnormal deposition, qPCR data showed the upregulated β-1,3-glucan synthase gene *fksA* and downregulated chitin synthase genes *chsB* and *chsD*, likewise, quantitative data revealed the increased β-1,3-glucan content and decreased chitin content in Δ*adhB* mutant. Totally, AdhB is important for maintaining normal distribution and synthesis of β-1,3-glucan and chitin for cell wall assembly.

K321 was identified as an important regulatory benzoylated site within AdhB protein, which is supported by the abolished Kbz signal of AdhB in K321R-HA and K321A-HA mutants. The alcohol dehydrogenase activity and phenotype of benzoylated point mutants (K to R or A) showed a strong similarity to that of the gene deletion mutants, including reduced alcohol dehydrogenase activity, lower seed colonization, decreased conidiation, increased aflatoxin production, and sclerotia formation, and more sensitive to cell wall damage. These suggested that disruption of benzoylated site in AdhB led to reduced enzymatic activity, then regulated the fungal morphogenesis, aflatoxin production, pathogenicity, and stress response in *A. flavus*. Given minor differences still exhibited between gene deletion and benzoylated point mutants, we speculated that this may be due to the presence of other sites with functional roles in AdhB. In addition, previous study showed that secondary structures of 2-hydroxyisobutyrylated Slt2 were noticeably different from those of unmodified Slt2 (35). 2-hydroxyisobutyrylation could increase the hydrophobic solvent-accessible surface area of Slt2, and then affect binding between the Slt2 enzyme and its substrates (35). Lack of the benzoylation of AdhB could decrease the thermal stability. Therefore, we speculated that the presence of benzoylation could affect the structure of AdhB protein, thereby influencing its enzyme activity, but proof of this would require further experimentation.

Since regulatory modification was physiologically meaningful, identification of the specific benzoyltransferase was important in benzoylation research. Emerging data classically discovered that histone acetyltransferase GcnE exhibits activities for acetylation and other acylations (1). For example, Gcn5-Ada2-Ada3 (ADA) complexes have the capacity to crotonylate histones (33), and GcnE mediates histone acetylation and benzoylation in yeast (8). In this study, we confirmed that GcnE was benzoyltransferase in *A. flavus*, and showed for the first time that AdhB is a direct benzoylation substrate of GcnE, and GcnE could regulate Adh enzymatic activity by affecting the benzoylation level of AdhB. Combining with our previous study that GcnE has histone acetyltransferase in *A. flavus* (31), it indicated that GcnE was major enzyme that adds Kbz and Kac in *A. flavus*. Repression of GcnE significantly inhibited the development and virulence, and reduced the Kbz and Kac levels in total proteins of *A. flavus*, but whether GcnE regulates crosstalk between Kbz and Kac needs further research. Moreover, the phenotype, Kbz and Kac levels of domain (GNAT and BRO) deletion and E139 point mutants showed a strong similarity to that of gene repression mutant. This may owe to that the conserved GNAT domain was the catalytic center of acyltransferase and E139 was the key catalytic site in GNAT domain, so these mutants also result in the loss of enzyme activity. GNAT domain also caused defect growth and decreased histone acetyltransferase activity in *C. albicans* (67). Conserved glutamate mutation could reduce acetylation, propionylation (68), and crotonylation rates (33), providing evidence that similar catalytic machinery was involved in the catalysis of different substrates. The bromodomain serves as a reader which recognizes acetylated lysine (1), and the Gcn5 bromodomain-mutant was defective in acetyl-lysine binding (69). In this study, the bromodomain deletion also leads to similar phenotype and Kbz level to *gcnE* repression mutant, suggesting that bromodomain also plays an important role in GcnE function in *A. flavus*.

Both sodium benzoate treatment and GcnE repression inhibit conidiation, sclerotia formation, and aflatoxin production, but they have opposite effects on benzoylation levels in *A. flavus*. Meanwhile, decreased benzoylation in AdhB causes decreased conidiation, increased sclerotia formation, and aflatoxin production. This inconsistency between the phenotype and the Kbz level change makes us to speculate that three possible reasons may exist for this discrepancy. First, the regulatory mechanisms of conidiation, sclerotia formation, and aflatoxin production were complex in *A. flavus*. Sodium benzoate, GcnE, and AdhB did not directly regulate the phenotype of *A. flavus*, but indirectly regulate by affecting the expression of some transcription factors. For example, sodium benzoate induction and *gcnE* deletion (31) both repress the transcription of genes within the aflatoxin cluster, whereas *adhB* deletion promotes their transcription, resulting in corresponding changes in aflatoxin production. Secondary, except for ADH, sodium benzoate, and GcnE may have other benzoylated targets in *A. flavus*. The regulation of benzoylation by sodium benzoate is global, while by *gcnE* is partial, so *gcnE* deletion cannot largely eliminate the benzoylation level in *A. flavus*. Third, benzoylation of different proteins may have distinct regulatory effects on the phenotype. Considering that there are no previous reports of benzoylation effect on non-histone protein, we used acetylation as a reference. For example, G6PD was negatively regulated by acetylation on K403 (53), but histone acetyltransferases hMOF was autoacetylated at an active site K274 residue to facilitate cognate substrate lysine binding and acetylation (70). Moreover, our previous studies have demonstrated that MYST family member MystA and HDAC family member Rpd3 negatively regulate aflatoxin biosynthesis (32, 71), but MYST family member MystB and HDAC family member HosA positively regulate aflatoxin biosynthesis (32, 71), which may also imply that different benzoyltransferase have different effects on phenotypes.

In conclusion, this work represents an expansion of our current study of Kbz in the development and pathogenicity of filamentous fungi, and lays the foundation for understanding of the diverse biological functions of lysine benzoylation in fungi.

## MATERIALS AND METHODS

### Strains and culture conditions

The *A. flavus* strains used in this study are listed in Table S2. Mutants were constructed by homologous recombination, and selectable marker (*pyrG*) was used for transformants selection (72). The mutant was verified by PCR, qPCR and sequencing. For growth and conidiation assays, *A. flavus* strains were cultured on YGT [20 g/L glucose, 5 g/L yeast powder, 22 g/mL ZnSO_4_·7H_2_O, 5 g/mL MnCl_2_·4H_2_O, 0.05 g/mL (NH_4_)_6_Mo_7_O_24_·5H_2_O, 11 g/mL H_3_BO_3_, 5 g/mL FeSO_4_·7H_2_O, 44.5 g/mL EDTA·2Na, 1.7 g/mL CoCl_2_·5H_2_O, and 1.6 g/mL CuSO_4_·5H_2_O] agar medium for 5 days at 37°C. Harvested conidia were counted by hemocytometer and microscope. For sclerotia formation assays, *A. flavus* strains were cultured on CM (10 g/L sucrose, 6 g/L yeast extract, and 6 g/L peptone) agar medium or YGT agar medium for 7 days at 37°C. For aflatoxin quantification, *A. flavus* strains were cultured on YES (15% source, 2% yeast extract, and 0.1% MgSO_4_·7H_2_O) liquid medium for 5 days at 29°C (71). The *gcnE*^xylP^ strain was cultured on YXT [20 g/L xylose, 5 g/L yeast powder, 22 g/mL ZnSO_4_·7H_2_O, 5 g/mL MnCl_2_·4H_2_O, 0.05 g/mL (NH_4_)_6_Mo_7_O_24_·5H_2_O, 11 g/mL H_3_BO_3_, 5 g/mL FeSO_4_·7H_2_O, 44.5 g/mL EDTA·2Na, 1.7 g/mL CoCl_2_·5H_2_O, and 1.6 g/mL CuSO_4_·5H_2_O] agar medium for conidia collection, then conidia was inoculated into YGT agar medium for protein extraction and Western blotting.

### Western blot

Proteins were extracted by RIPA lysis buffer (Beyotime, P0013B), and separated on sodium dodecyl sulfate polyacrylamide gel electrophoresis (SDS-PAGE), then transferred onto polyvinylidene fluoride membrane (Millipore, IPVH00010) with a Bio-Rad electroblotting apparatus. Subsequent transformants were subjected to Western blotting analysis with mouse pan anti-Kbz (PTM Biolabs, PTM-762), pan anti-Kac (PTM Biolabs, PTM-102), pan anti-Ksucc (PTM Biolabs, PTM-401), anti-actin (Servicebio, GB113225) or anti-HA (CST, 3724S) primary antibody, and secondary antibody (Thermo, 31460 and 31430). The Ultra High Sensitivity ECL Kit was used for Western blot detection (Glpbio, GK10008), and photographed in GBox XT4 Chemiluminescence and Fluorescence Imaging System. Quantification of WB data were performed using the Fiji software.

### Aflatoxin assay

For quantifying the aflatoxin production, 10^4^ conidia suspension or mycelial plugs (5 mm diameter) of strain were cultured on YGT or YES liquid medium in the dark at 29°C for 6 days. Aflatoxin was extracted in addition of an equal volume of dichloromethane into the liquid medium and shaken for 30 min at room temperature. Dichloromethane layer was collected and dried in the fume hood, then mycelium was collected, and the dry weight was measured. The extracted aflatoxin was resuspended in dichloromethane (1 mL/mg mycelia). Finally, aflatoxin B_1_ was detected and quantified by thin-layer-chromatography (TLC) (31). Each experiment was performed with five replicates three times.

### Stress response assay

The susceptibilities of the gene disruption mutants to cell wall stress agent Conge red (500 µg/mL CR), hyperosmotic stress mediator sodium chloride (1.2 M NaCl), genotoxic agent methyl methanesulfonate (0.01% MMS), oxidative stress agent hydrogen peroxide (3.5 mM H_2_O_2_) and sodium benzoate (30 mM) were tested on YGT medium at 29°C for 5 days (31). Each strain was examined on five plates, and each experiment was repeated three times.

To test sensitivity to cell wall perturbing agents, equal conidia were inoculated onto YGT medium containing calcofluor white 28 (50–400 μg/mL CFW) and congo red (62.5–500 μg/mL CR) for 5 days. For staining the glucan and chitin components of the cell wall, CR and CFW were, respectively, used at a final concentration of 10 mg/mL to stain the fungal hyphae for 5 min at room temperature. After washed three times with phosphate-buffered saline (PBS), the mycelium was observed and imaged with florescence microscope.

### Quantification of β-1,3-glucan and chitin

Fresh conidia of *A. flavus* were inoculated into YGT medium and incubated at 37°C for 1 day. Mycelium was collected and ground in liquid nitrogen for the determination of chitin and glucan. β-1,3-Glucan and chitin content was examined using the aniline blue assay (73) and acid hydrolysis assay (74), as previously described.

### Benzoylome proteomics assay

Mycelia grown in YGT liquid media were harvested and rinsed with cold PBS buffer three times, then grinded by liquid nitrogen. Proteins were extracted by lysis buffer (8 M urea, 1% Triton-100, 10 mM dithiothreitol, and 1% protease inhibitor cocktail), and re-dissolved in 8 M urea. After trypsin digestion, the tryptic peptides were fractionated by high pH reverse-phase HPLC with Agilent 300Extend C18 column (5 µm particles, 4.6 mm ID, and 250 mm length). To enrich benzoylated peptides, tryptic peptides were dissolved in NETN buffer (50 mM Tris-HCl, 100 mM NaCl, 1 mM ethylenediaminetetraacetic acid, and 0.5% NP-40, pH 8.0) and then incubated with anti-benzoyllysine antibody agarose beads at 4°C overnight. Subsequently, the beads were washed four times with NETN buffer and two times with sterilized water. The bound peptides were eluted with 0.1% trifluoroacetic acid for liquid chromatography-tandem mass spectrometry (LC-MS/MS) analysis, as previously described (16).

The resulting MS/MS data were identified using MaxQuant (v.1.5.2.8) against the NCBI_*A. flavus* protein database and concatenated with reverse decoy database. Two or fewer missed cleavages were allowed for trypsin/P. Mass tolerance of precursor ions was 20 ppm in the first search and 5 ppm in the main search, and the mass tolerance of fragment ions was 0.02 Da. False discovery rate thresholds for peptides, proteins, and modification sites were adjusted to <1%, and the minimum score for peptides was set >40.

### Validation of benzoylated proteins and sites *in vivo*

We mutated lysine to arginine or alanine and confirmed its HA fusion mutations by PCR and WB. For IP, anti-HA magnetic beads (MCE, HY-K0201) were washed three times with PBST, and incubated with whole-cell lysates overnight at 4°C. Then, the beads were washed three times with PBST to remove unbound proteins, and the bound proteins were boiled in 1× protein loading buffer for 5 min. Subsequently, anti-HA antibody and pan anti-Kbz antibody were used for immunoblotting.

### *In vivo* and *in vitro* alcohol dehydrogenase activity assay

The enzyme activity of AdhB was performed according to a reference method with some modifications (60). For Adh activity assay, strain was grown in YES liquid medium at 37°C for 24 h. Mycelium was harvested and rinsed three times with distilled water, then immediately frozen in liquid nitrogen and weighed. After adding 1 mL of extraction buffer (100 mM KH_2_PO_4_, 2 mM MgCl_2_, and 1 mM DTT), the samples were two times vortex and then placed on ice for 5 min. After centrifugation (13,000 revolutions/min, 20 min, 4°C), the cell-free extracts were transferred to a new tube, and enzymatic activity was measured according to kit instructions (Solarbio, BC1085).

The full-length cDNA of AdhB, AdhB^K321R^, and AdhB^K321A^ from *A. flavus* was cloned into the pET-32a to purify proteins in *E. coli* Rosetta (DE3). The primers used are listed in Table S3. The detailed protein purification protocol used in this study has been previously described (75). The final purified proteins were verified by CBB stain and WB following sodium dodecyl sulfate-polyacrylamide gel electrophoresis (SDS-PAGE). The AdhB and site-mutated proteins were used for alcohol dehydrogenase activity assay *in vitro*, and enzymatic activity was also measured according to kit instructions (Solarbio, BC1085).

### Thermal stability assay

For thermal stability between AdhB and their mutants, the Nano-DSC instrument (Calorimetry Sciences Corp., Lindon, UT, USA) was used to measure the melting temperature of phosphatase *in vitro*. The detailed protocol used in this study has been previously described (76). DSC analysis software was used for curve fitting and data analysis.

### Acetaldehyde determination

Acetaldehyde determination was performed as described previously with minor modifications (57). For acetaldehyde determination, 25 µL of sample solution was mixed thoroughly with 300 µL Tris-HCl buffer (1 M, pH 8.0) and 25 µL NAD^+^ solutions (10 mM). Acetaldehyde dehydrogenase (Macklin, A875363) was added to the mixture and then incubated at 25°C for 30 min. The absorbance of the reaction mixture was read at 340 nm.

### Infection assay

The infection assay of *A. flavus* was carried out according to previous study (32). Viable maize seeds were washed with 0.05% sodium hypochlorite and 75% ethanol, and rinsed three times with sterilized water, then inoculated with 10^6^ spores of each respective strain and cultured in the dark at 29°C for 5 days. Meanwhile, peanuts and maize seeds were inoculated with sterilized water for control. During the infection period, suitable volume of sterile water was added to maintain the moisture of the filter paper. After inoculation, maize seeds were harvested to count conidia, and quantify aflatoxin and fungal burden, and the experimental process refers to our previous experimental methods (32).

### Penetration assay

The fungal penetration assay refers to the previous study (77) with a slight modification. Fresh conidia were inoculated onto cellophane overlaying CM and YGT medium, and cellophane was removed from the surface of the plates following incubation for 4 days, then these plates were maintained for 1 day to observe colonies diameter. Each experiment was repeated three times.

### Determination of fungal growth in maize medium

Fresh corn kernels (250 g/L) were homogenized, and the filtrate was collected through gauze, and then 1.5% agar powder was added and autoclaved to obtain maize medium. 10^3^ conidia suspension of strain was inoculated onto the plates of maize medium at 37°C for 5 days, then colony diameters were measured.

### Lipase activity assay

Lipase activity assays were executed according to previous studies (78).10^3^ conidia were inoculated on YGT solid medium and YGT solid medium supplemented with 0.3% triglyceride. Plates were incubated at 37°C for 4 days and then the growth inhibition rate was calculated.

### *In vitro* benzoylation assay

The AflGcnE full-length DNA was cloned into pET-32a and then the vector was transformed into the *E. coli* Rosetta (DE3) strain. This strain was cultured in 1 L Luria-Bertani medium containing ampicillin and chloramphenicol for 4 h, then IPTG was added with subsequent incubation at 16°C for 18 h. After harvesting, the cell pellets were washed and resuspended in binding buffer A (50 mM Tris-HCl, 500 mM NaCl, and 20 mM imidazole, pH 7.4) and disrupted with ultrasound to extract protein. The fusion protein with His-tag was eluted with a gradient concentration of imidazole buffer (75), and finally with the eluent buffer (50 mM Tris-HCl, 500 mM NaCl, and 300 mM imidazole, pH 7.4). Benzoylation assay was performed based on a previously described method (33, 35). The total protein of *A. flavus*, 300 µM benzoyl-CoA and purified recombinant GcnE-His protein were incubated in a reaction buffer (50 mM Tris-HCl, pH 7.5, 100 mM NaCl, 1 mM EDTA, and 1 mM DTT), and then incubated at 30°C for 4 h. The reaction products were analyzed by SDS-PAGE and Western blotting using pan anti-Kbz antibodies.

### qPCR assay

Total RNA was isolated from the mycelia of each strain with TRIzol reagent (32), and cDNA was synthesized with First Strand cDNA Synthesis Kit (Vazyme, R312-01). The qPCR analysis was carried out on Real-time PCR PikoReal 96 System with qPCR SYBR Green Master Mix (Yeasen, 11201ES08). The standard curve method was used to analyze relative quantification, and *actin* was used as housekeeping gene. All qPCR primers are listed in Table S4. Each experiment was repeated three times.

### Statistical analysis

Data were presented as means ± standard deviations (SD) of at least three biological replicated samples in figures. SPSS 19.0 and GraphPad Prism 8.0 were used for data analysis. Significance was evaluated by one-way analysis of variance (ANOVA) using LSD test, and *P* value < 0.05 was considered to indicate a significant difference.

## Data Availability

All the raw data of MS/MS were uploaded to the China National Gene Bank database with the identifier CNP0003875.

## SUPPLEMENTAL MATERIAL

Supplemental material mbio.02665-24-s0001.pdf Fig. S1 to S21; Tables S1 to S4.

ASM does not own the copyrights to Supplemental Material that may be linked to, or accessed through, an article. The authors have granted ASM a non-exclusive, world-wide license to publish the Supplemental Material files. Please contact the corresponding author directly for reuse.
