# Supplementary material for: Importance of benzoyltransferase GcnE and lysine benzoylation of alcohol dehydrogenase AdhB in pathogenesis and aflatoxin production in Aspergillus flavus
Source: mBio. 2024 Nov 27;16(1):e02665-24. doi: 10.1128/mbio.02665-24 (PMC11708022; doi:10.1128/mbio.02665-24)
Supplement: Supplemental material — Fig. S1 to S21; Tables S1 to S4. [file mbio.02665-24-s0001.pdf]

Supporting information

Fig S1. Identification of lysine benzoylation in *A. flavus*.

(A) Western blot analysis of Kbz in *Escherichia coli* (1), *Saccharomyces cerevisiae* (2), *Arabidopsis* (3), *Danio rerio* (4), *Mus musculus* (5) and RAW cells (6). (B) Western blot analysis of Kbz in *A. flavus* (7), *A. fumigatus* (8), *A. nidulans* (9) and *Saccharomyces cerevisiae* (2). (C) Western blotting analysis of acetylated proteins in response to sodium benzoate (SB) with the indicated concentration in *A. flavus*. (D) Relative Kbz and Kac levels were quantified and normalized to total protein (n=3). Asterisks represent statistically significant differences ($P < 0.05$). (E) Colony morphology and sclerotium formation of *A. flavus* with sodium benzoate treatment at different concentration. (F-H) Colony diameters analysis (F), conidia production analysis (G) and sclerotia production analysis (H) of *A. flavus* under SB treatment (n=3). (I) Aflatoxin B_1 production of *A. flavus* with sodium benzoate treatment at different concentration (n=3). The little letters above the columns show significant differences ($P < 0.05$).

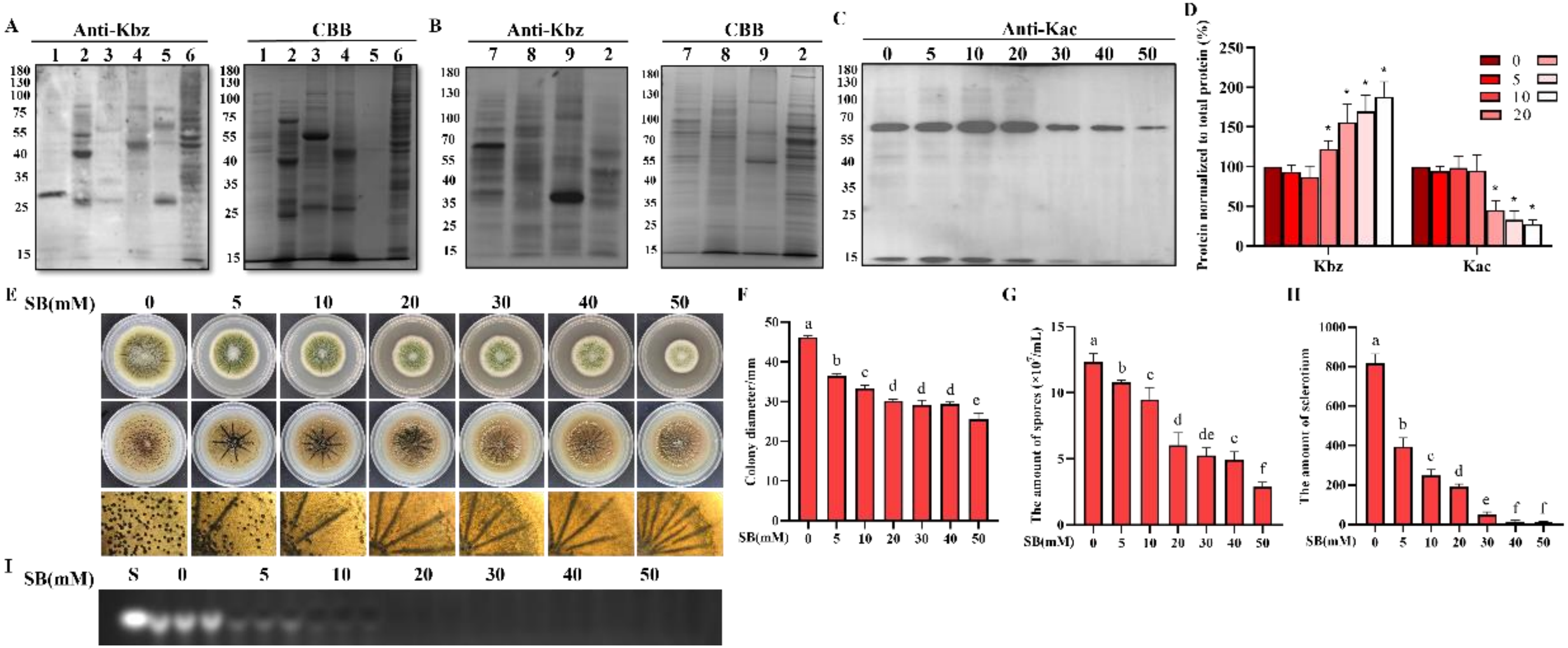
A
Anti-Kbz
CBB
B
Anti-Kbz
CBB
C
Anti-Kac
D
Protein normalized to total protein (%)
Kbz
Kac
E
SB(mM)
F
Colony diameter/mm
G
The amount of spores (×10⁷/mL)
H
The amount of sclerotium
I
SB(mM)

14

Fig S2. Effects of different stresses on phenotype and acetylation of *A. flavus*.

(A) Profile of lysine acetylation in *A. flavus* under different stress conditions. (B) Relative Kbz and Kac levels were quantified and normalized to total protein (n=3). (C) Morphology of WT under 0.01% MMS, 1.2 M NaCl, 500 μg/mL CR, 3.5 mM H_2O_2 or 30 mM SB. (D-E) Quantification of the colony diameter (D) and conidia production (E) under different stress (n=3). Asterisks represent statistically significant differences ($P < 0.05$).

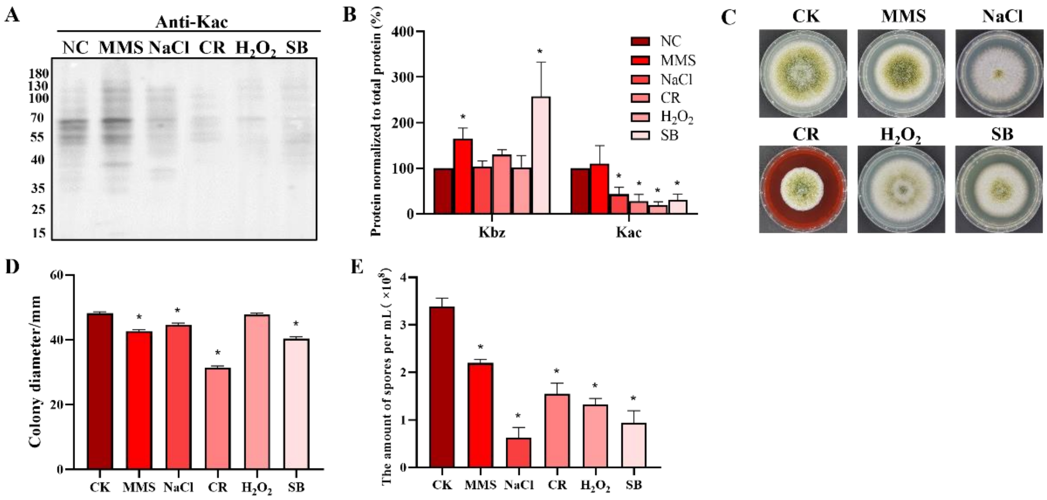

Fig S3. Basic analysis of benzoylomic data.

(A) Workflow for lysine benzoylome analysis of *A. flavus*. (B) Length distribution of tryptic peptides. (C) Distribution of peptide score and mass error. (D) Distribution of benzoylated proteins based on the number of modified sites. (E) The amino acid sequence pattern surrounding the benzoylated lysine. The feature sequence for modified site and its enrichment statistics were analyzed by MoMo software. The intensity map shows the relative abundance for ± 10 amino acids from the lysine-benzoylated site. Red indicates that this amino acid is significantly enriched near the modification site, and green indicates that this amino acid is significantly reduced near the modification site.

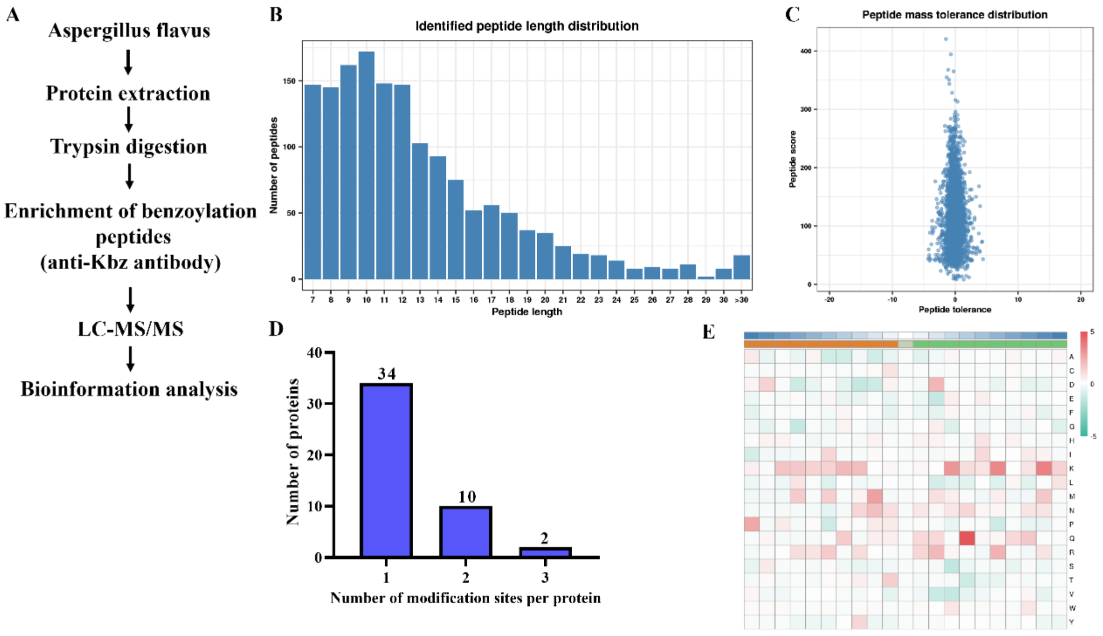

Fig S4. GO and KEGG annotation and enrichment analysis of benzoylated proteins.

(A) GO annotation analysis of benzoylated proteins. (B-D) Cellular components (B), biological processes (C) and molecular functions (D) enrichment analysis of benzoylated proteins (p-value<0.05). (E) KEGG annotation of benzoylated proteins.

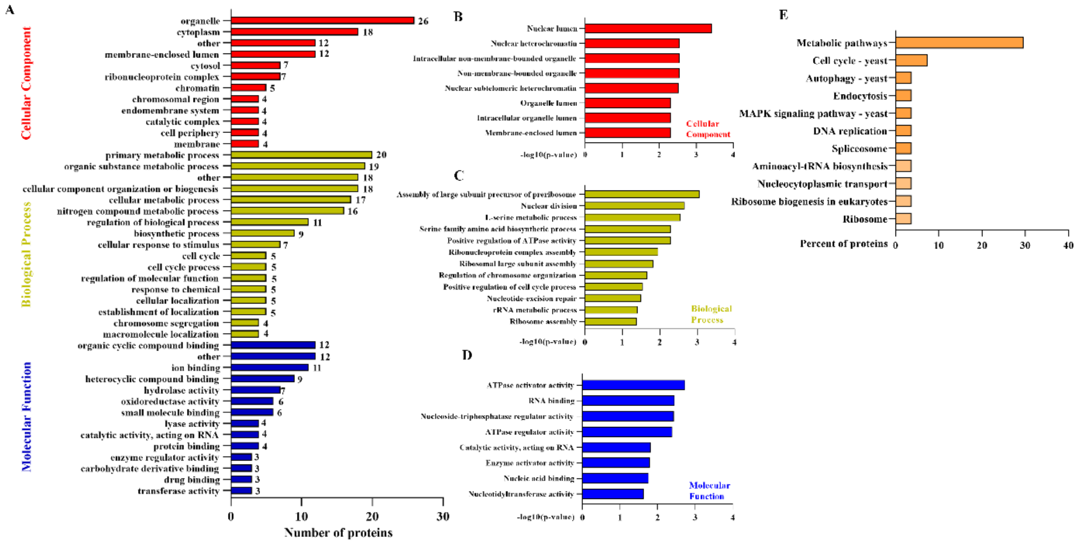

Fig S5. Crosstalk between the benzoylated proteins and acetylation- and succinylation-modified proteins.

(A) Statistical analysis of acetylated, succinylated and benzoylated modified proteins and sites in *A. flavus*. (B-C) Venn diagrams show lysine benzoylated, acetylated and succinylated proteins (B) and sites (C) analysis of *A. flavus*. (D) The co-modified proteins of benzoylation and acetylation.

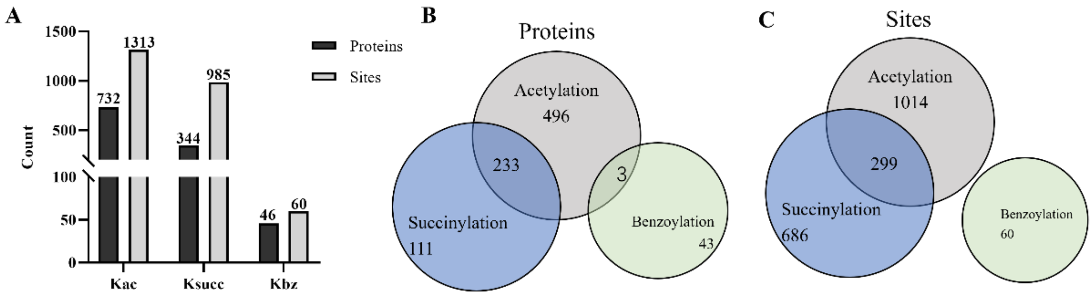

D

Protein	Position	Modified sequence
FoxA	425/433	VTK(bz)AAWPYMLK(bz)QK
	477	TLAIEGAK(ac)YNIK
	839	HVYSK(ac)FGAYK
PLD	107/110	PQGK(bz)PPK(bz)EDK
	39	MLK(ac)ETYK
P450	325/328	IPK(bz)GTK(bz)IIIPQR
	171	LPSFLAK(ac)HATQLR

Fig S6. A representative MS/MS spectrum of a benzoylated peptide from the alcohol dehydrogenase B (AdhB).

Raw file	Scan number	Mass analyzer	Score	m/z	Proteins
KD031PIBZ_IP2_2	9577	FTMS	47.603	769.377	B8NFP9

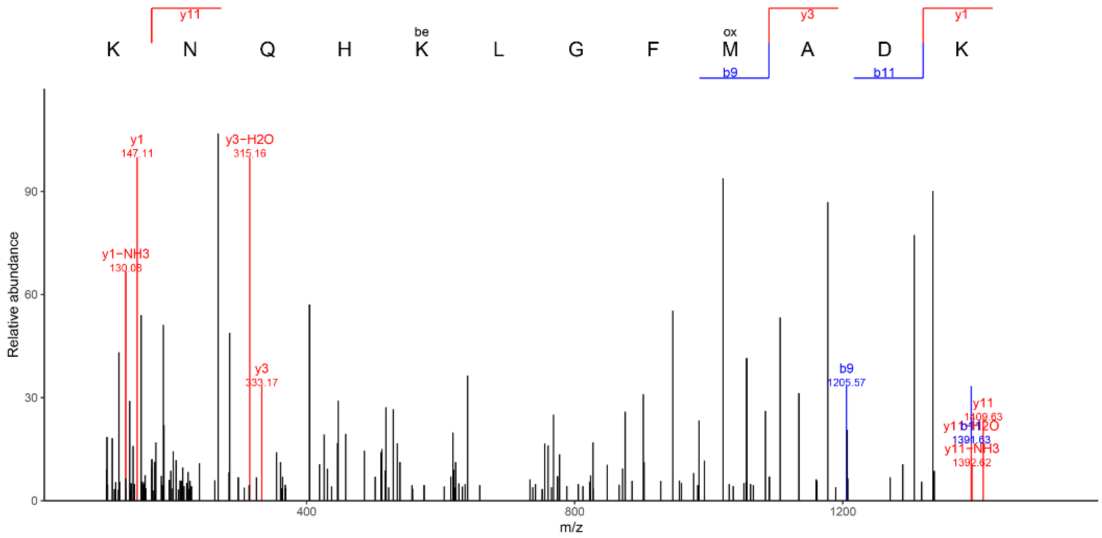

Fig S7. Construction and confirmation of *adhB* mutants.

(A) Sequencing confirmation of K321-HA, K321R-HA and K321A-HA strains. (B) Relative *adhB* levels protein was quantified and normalized to Actin (n=3). (C-D) Genomic PCR (C) and qPCR (D) verification of Δ*adhB* and *adhB*-com. (E) Sequencing confirmation of *adhB*^{K321R} and *adhB*^{K321A} mutants. Asterisks represent statistically significant differences (P < 0.05).

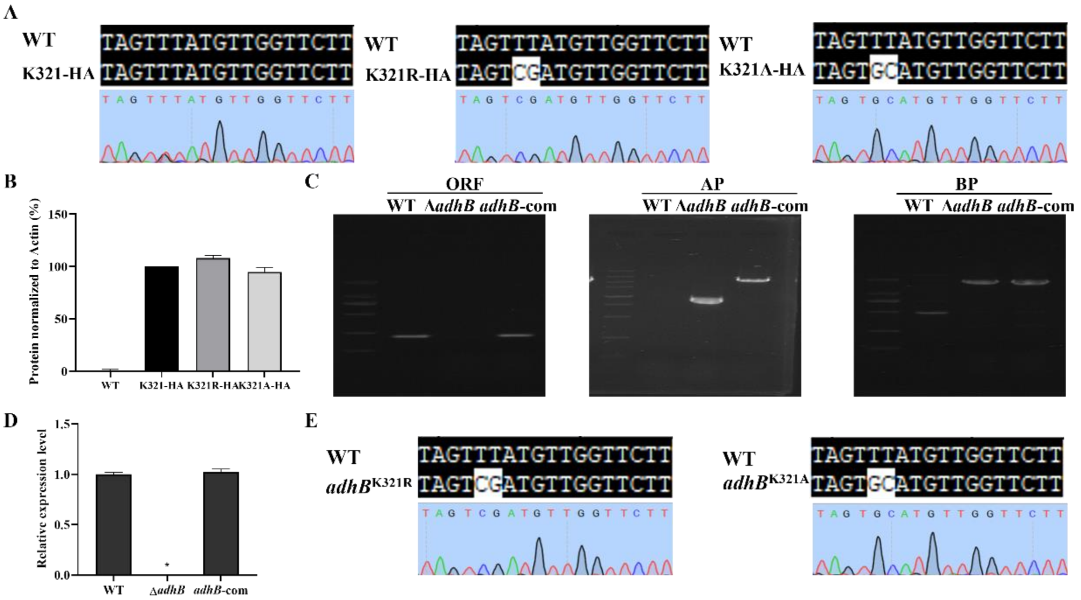

Fig S8. AdhB is not an acetylated or succinylated protein.

(A) Benzoylation, acetylation and succinylation of AdhB was verified by using immunoprecipitation and western blotting. (B) Structural modeling of AdhB.

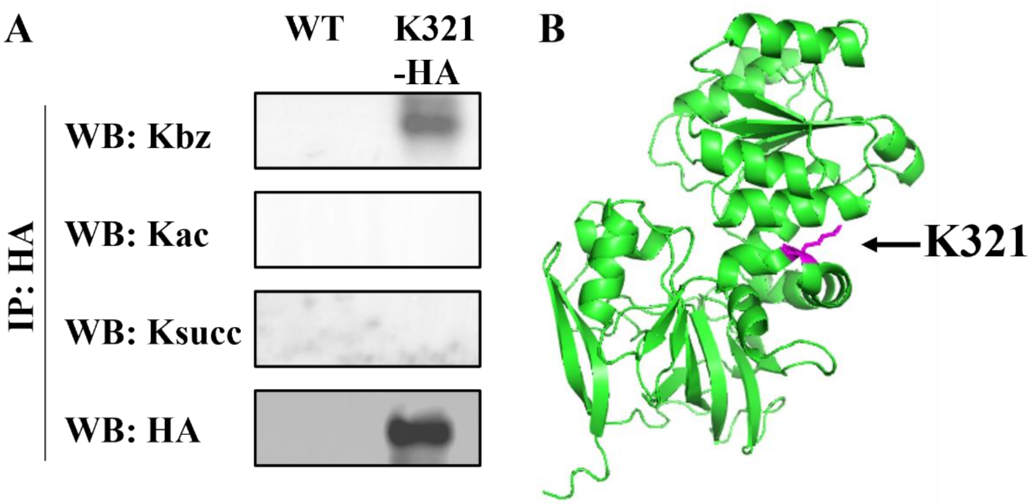

Fig S9. Thermal stability of AdhB, AdhB^{K321R} and AdhB^{K321A} protein.

(A) Purification of AdhB, AdhB^{K321R} and AdhB^{K321A} protein using Ni-NTA column chromatography. (B) Western blot analysis of His in purified AdhB, AdhB^{K321R} and AdhB^{K321A} protein. (C) Thermal stability of AdhB and their mutant proteins *in vitro*. Purified protein samples were measured for melting temperature with Nano differential scanning calorimetry (Nano-DSC) instrument, and curve fitting and data analysis were performed with Nano-DSC Analysis System.

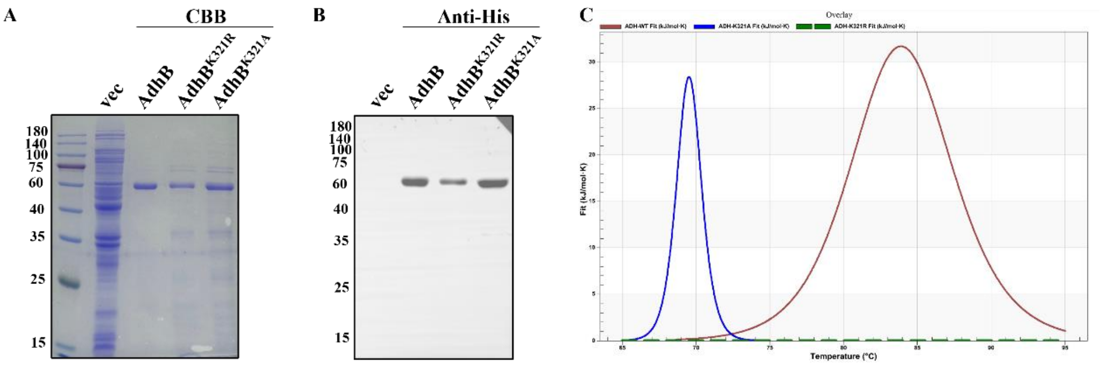

Fig S10. AdhB is required for conidiation.

(A) Expression level of *adhB* gene on various development stages in *A. flavus*. The little letters above the columns show significant differences (P < 0.05). (B) Colony morphology of WT and *adhB* mutants grown on glycerol media at 37°C for 5 days. (C) Colony morphology of WT and *adhB* mutants grown on YGT media at 37°C for 5 days. (D) Microscopic observation of the conidiophores morphology for different strains. Bar=20 μm. (E) Colony diameter by the different strains on glycerol and YGT media. (F) Conidia production by the different strains on glycerol and YGT media. (G) Transcriptional expression levels of *abaA* and *brlA* genes (n=3). Asterisks represent statistically significant differences (P < 0.05).

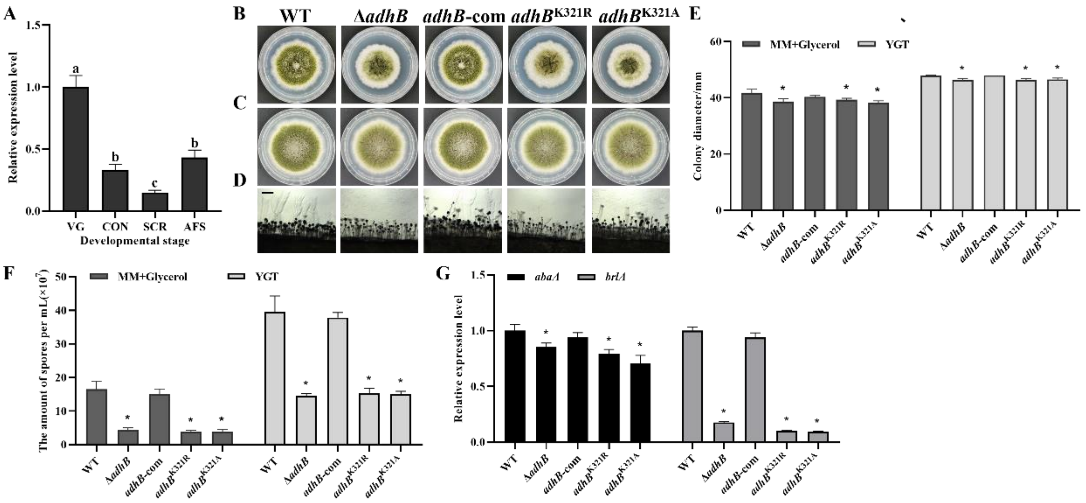

Fig S11. AdhB is important for sclerotia formation.

(A) Phenotype of WT, Δ*adhB*, *adhB*-com, *adhB*^{K321R} and *adhB*^{K321A} strains on CM media, after grown for 7 days at 37°C. (B) Amount of sclerotia produced by different strains on CM media (n=3). (C) Transcriptional expression levels of *nsdC* and *sclR* genes (n=3). Asterisks represent statistically significant differences (P < 0.05).

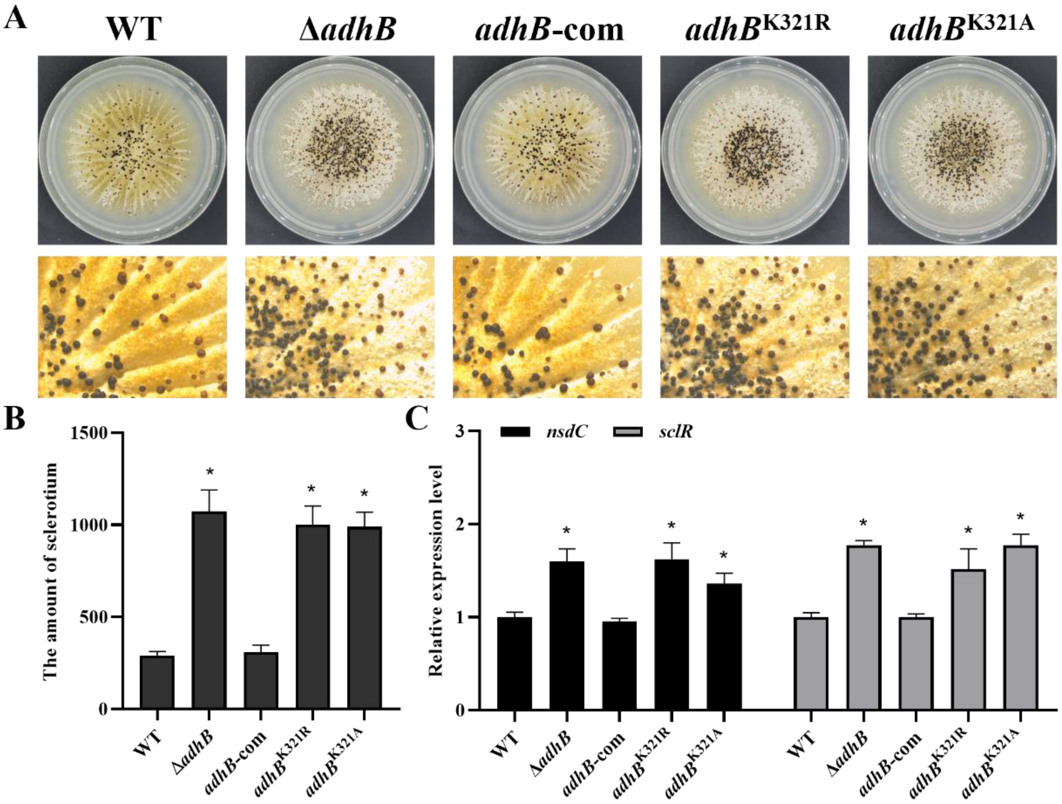

Fig S12. Quantification of conidia and AFB_1 production.

(A) TLC assay was used to detect the AFB_1 production extracted from the infected maize seeds (n=3). (B) Quantification of conidia from the infected maize seeds (n=3). (C) Quantification analysis of AFB_1 in TLC results by optical density (n=3). Asterisks represent statistically significant differences ($P < 0.05$).

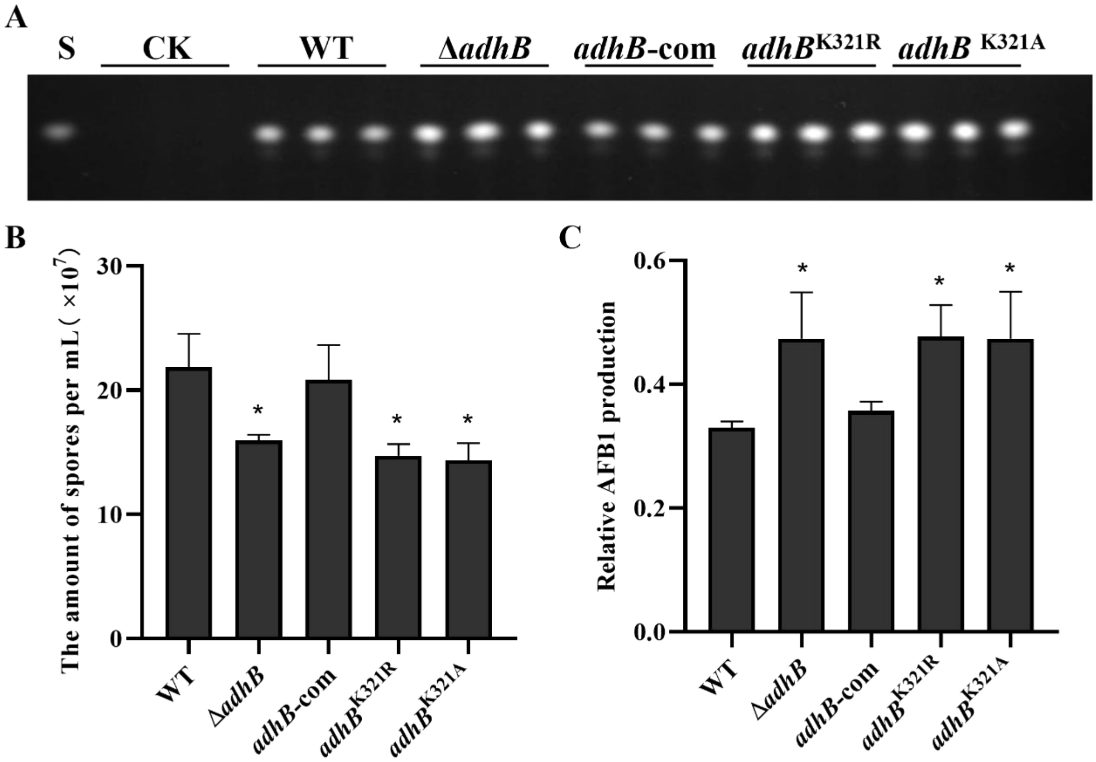

Fig S13. Roles of AdhB in fungal penetration and lipase activity.

(A) WT and *adhB* mutants were grown on YGT media overlaid with a cellophane layer for 4 days (above), and the plates were further incubated for 1 day after removing the cellophane layer (below). (B) Statistical analysis of colony diameter in YGT media (n=3). (C) Morphology of WT and *adhB* mutants under 0.3% glyceryl tributyrate. (D) The inhibition rate of WT and *adhB* mutants under glyceryl tributyrate treatment (n=3). Asterisks represent statistically significant differences ($P < 0.05$).

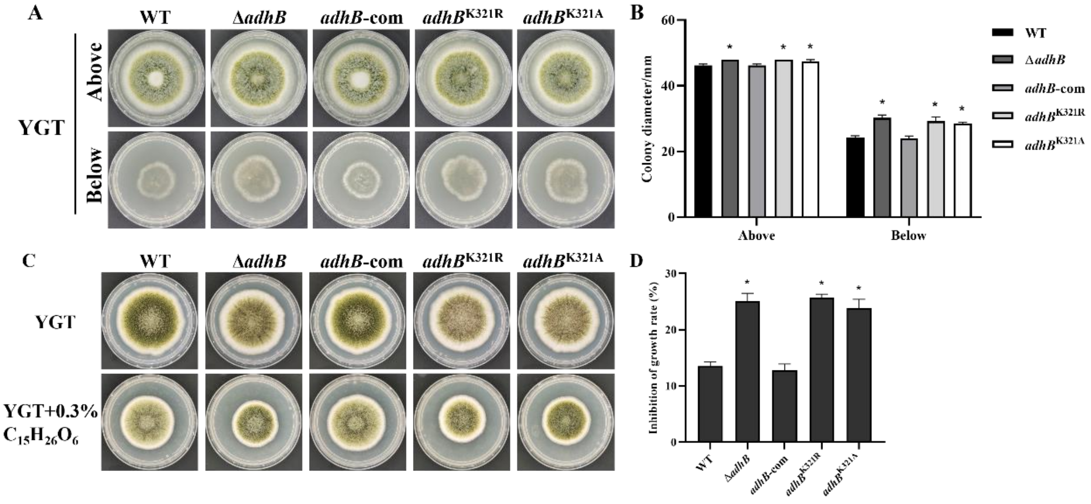

Fig S14. The inhibition rate of *adhB* mutants under CR and CFW treatment.

(A) Morphology of WT, Δ*adhB* and *adhB*-com under cell wall perturbing agents (62.5-500 μg/mL CR and 50-400 μg/mL CFW). (B-C) Growth inhibition rate of WT, Δ*adhB* and *adhB*-com strains under CR (B) or CFW (C) (n=3). Asterisks represent statistically significant differences ($P < 0.05$). (D) Morphology of WT, Δ*adhB*, *adhB*-com, *adhB*^{K321R} and *adhB*^{K321A} under cell wall perturbing agents (500 μg/mL CR and 400 μg/mL CFW).

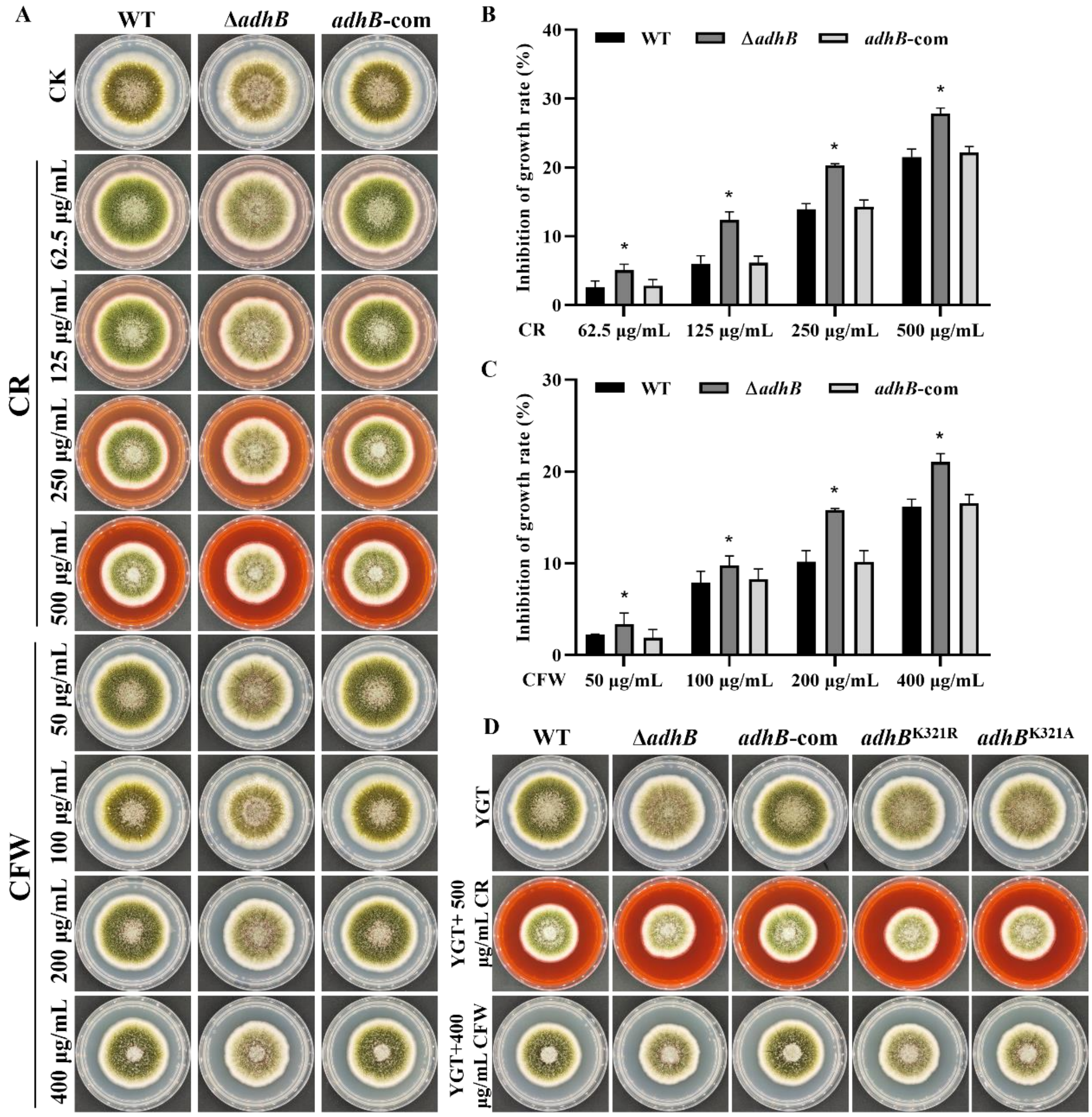

Fig S15. Construction and confirmation of Δ*hatA* and *gcnE*^{xylP} strains.

(A) The strategy for constructing Δ*hatA* and *gcnE*^{xylP} mutants. (B) Genomic PCR verification of Δ*hatA* mutant. (C) Genomic PCR verification of *gcnE*^{xylP} mutant. (D) Colony morphology of WT and *gcnE*^{xylP} strains grown on GMM and XMM media at 37°C for 4 days.

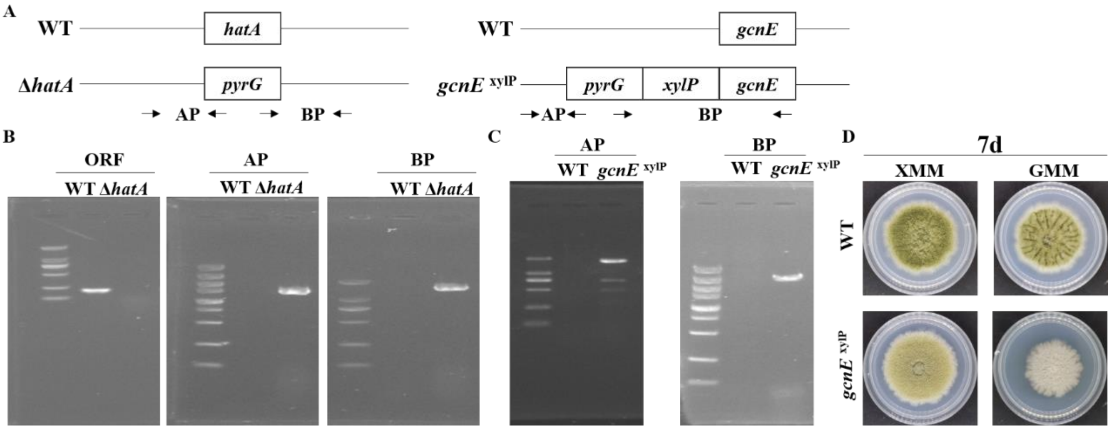

Fig S16. Classical acyltransferase has acetyltransferase activity.

(A) Western blot analysis of Kac in WT, Δ*mystA*, Δ*mystB*, Δ*rtt109*, Δ*gcnE* and Δ*hatA* strains. (B) Relative Kbz and Kac levels were quantified in WT, Δ*mystA*, Δ*mystB*, Δ*rtt109*, Δ*gcnE* and Δ*hatA* strains (n=3). (C) Western blot analysis of Kac in WT, $gcnE^{xylP}$, Δ*ada2,* Δ*ada3*, $gcnE^{xylP}$-Δ*ada2* and $gcnE^{xylP}$-Δ*ada3* strains. (D) Relative Kbz and Kac levels were quantified in WT, $gcnE^{xylP}$, Δ*ada2,* Δ*ada3*, $gcnE^{xylP}$-Δ*ada2* and $gcnE^{xylP}$-Δ*ada3* strains (n=3). Asterisks represent statistically significant differences (P < 0.05).

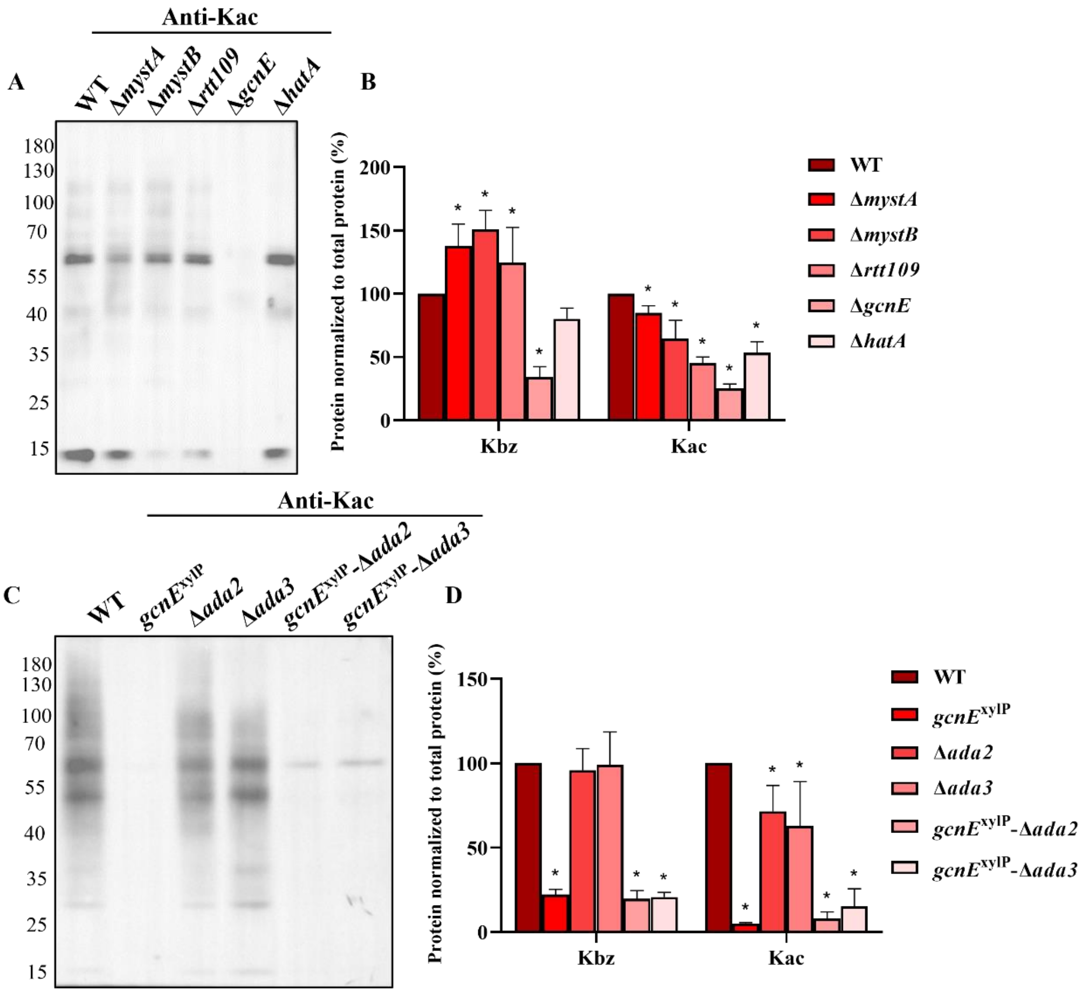

Fig S17. Benzoylation level of *gcnE*^{xylP} mutant under various inductive conditions in *A. flavus*. Western blot analysis of Kbz in WT and *gcnE*^{xylP} mutant. Xylan, xylose or glucose was used as carbon sources, respectively.

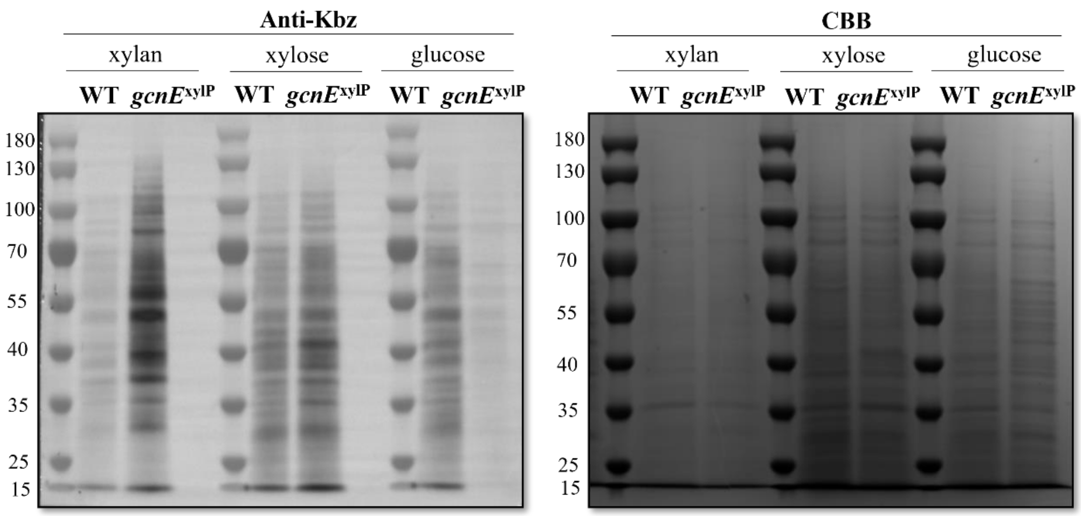

Fig S18. Construction and confirmation of ADA complex and *gcnE* deletion mutants.

(A) Genomic PCR verification of Δ*ada2*, Δ*ada3, gcnE*^{xylP}-Δ*ada2, gcnE*^{xylP}-Δ*ada3* and *gcnE*^{xylP}-*adhB*-HA mutants. (B) WB verification of *gcnE*^{xylP}-*adhB*-HA strain. (C) Schematic drawing of *gcnE* domain mutants. (D) Genomic PCR verification of *gcnE*$^{\Delta BRO}$ and *gcnE*$^{\Delta GNAT}$ strains. (E) Sequencing confirmation of *gcnE*^{E139H} strain. (F) Relative expression level of *gcnE* gene in *gcnE* mutants (n=3). Asterisks represent statistically significant differences ($P < 0.05$).

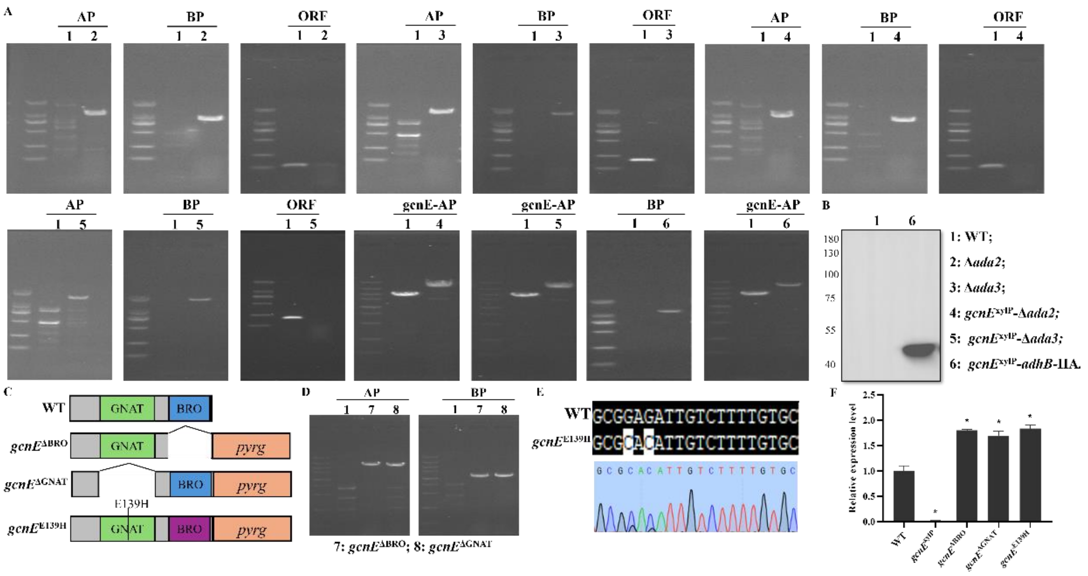

Fig S19. Purification and structural simulation of GcnE protein.

(A) Purification of GcnE using Ni-NTA column chromatography. M: protein marker; Line 1: sediment of protein; Line 2: supernatant of protein; Line 3: flow-through fraction from Ni-NTA Column; Line 4-6: elution fractions with Buffer B, C, D, respectively; Line 7-14: elution fractions with Buffer E. (B) Structural modeling of GcnE.

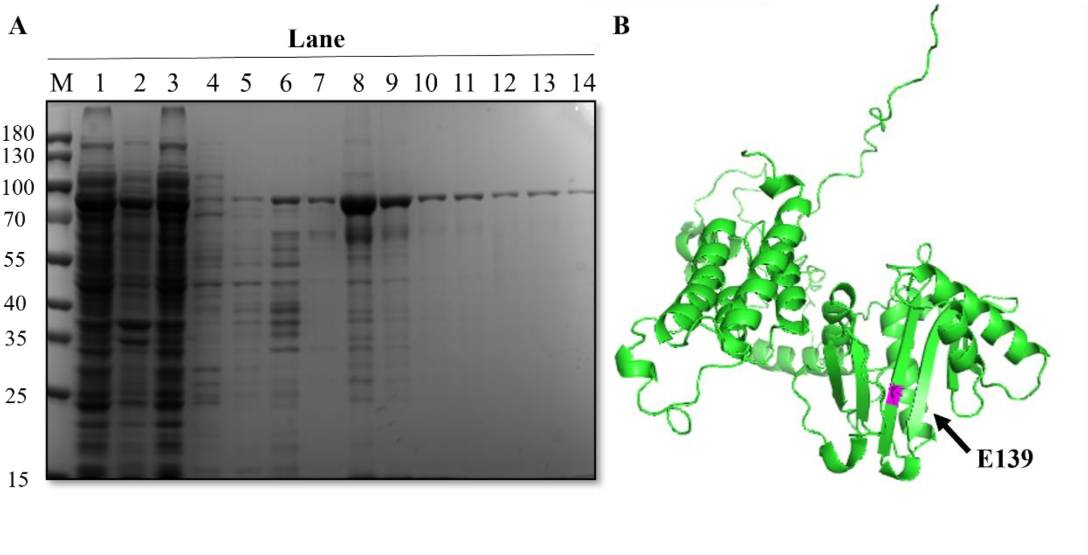

Fig S20. E139 in GNAT domain is pivotal for GcnE biological function.

(A) Schematic drawing of the functional domains and catalytic site of GcnE in *A. flavus*. (B) Western blot analysis of Kbz and Kac in WT, *gcnE*^{xylP}, *gcnE*$^{\Delta BRO}$, *gcnE*$^{\Delta GNAT}$ and *gcnE*^{E139H} strains. (C) Relative Kbz and Kac levels were quantified in *gcnE* mutants (n=3). (D) The Adh enzymatic activity in *gcnE* mutants (n=3). (E) Phenotypic characterization of WT, *gcnE*^{xylP}, *gcnE*$^{\Delta BRO}$, *gcnE*$^{\Delta GNAT}$ and *gcnE*^{E139H} strains grown on YGT media. (F) Colony diameters analysis of WT and *gcnE* mutants (n=3). (G) Amount of conidia produced by WT and *gcnE* mutants on YGT media (n=3). (H) Amount of sclerotia produced by WT and *gcnE* mutants on YGT media (n=3). (I) TLC assay of AFB$_1$ production by the WT and *gcnE* mutants cultured in YGT liquid media (n=3). S indicates AFB$_1$ standard. (J) Quantification analysis of AFB$_1$ in TLC results by optical density (n=3). Asterisks represent statistically significant differences ($P < 0.05$).

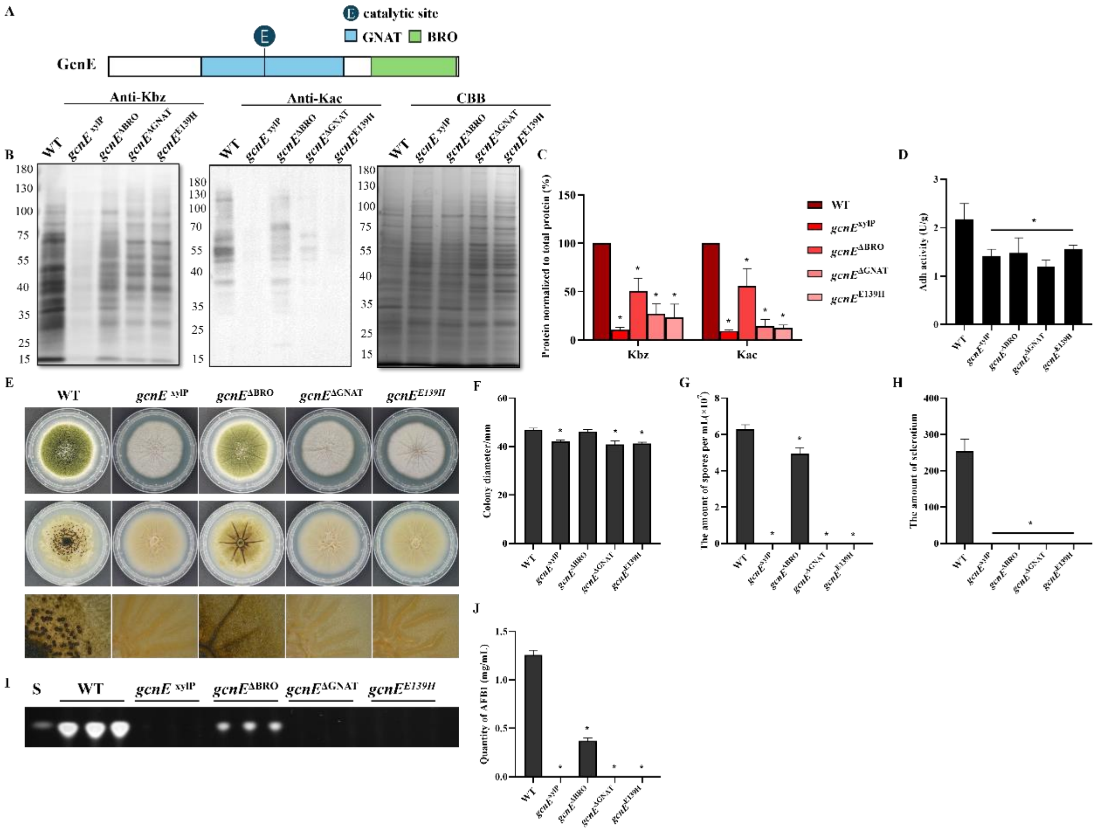

Fig S21. E139 site in GNAT domain is involve in stress response.

(A) Morphology of WT, *gcnE* ^{xylP}, *gcnE*$^{\Delta BRO}$, *gcnE*$^{\Delta GNAT}$ and *gcnE*^{E139H} strains under cell wall stress by 500 µg/ml CR and DNA damage stress by 0.01% MMS. (B) Growth inhibition of WT and *gcnE* mutants under cell wall and DNA damage stress. Asterisks represent statistically significant differences ($P < 0.05$).

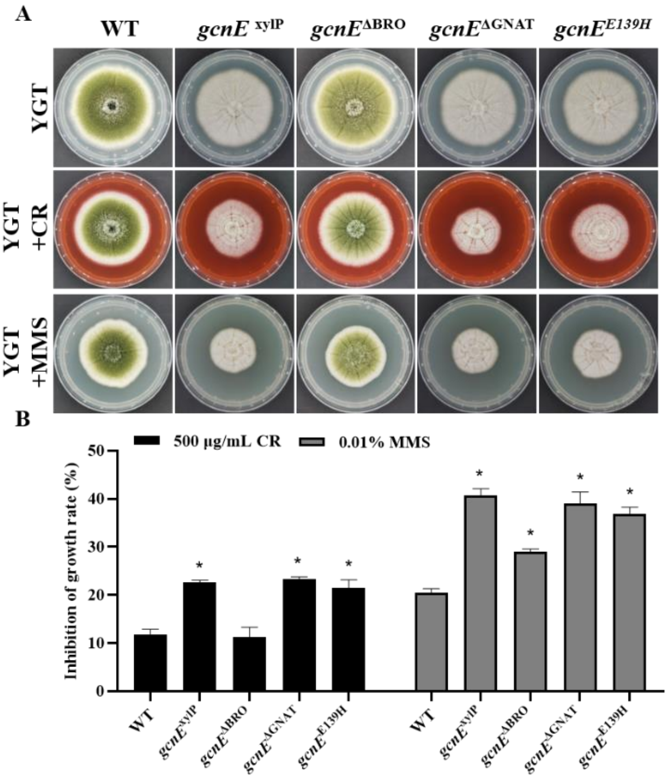

Table S1. The benzoylated sites in *Aspergillus flavus*.

Classification	Protein accession	Position	Modified sequence	Protein description
Enzyme	B8MZH7	63	RDTAETTQSK(bz)NK(bz)K	RNA helicase
	B8MZH7	61	RDTAETTQSK(bz)NK(bz)K	RNA helicase
	B8N242	456	DTIMTK(bz)R	GTP1/OBG protein
	B8N2E2	33	SELK(bz)RRQK(bz)LR	Lysine--tRNA ligase
	B8N2E2	37	SELK(bz)RRQK(bz)LR	Lysine--tRNA ligase
	B8N2W3	25	ITMLK(bz)K(bz)RK	Nucleolar RNA methyltransferase Nop2
	B8N2W3	26	ITMLK(bz)K(bz)RK	Nucleolar RNA methyltransferase Nop2
	B8N4D6	498	YYVQK(bz)NWK	Ubiquitin C-terminal hydrolase
	B8N900	304	WNK(bz)NDMAIWDNR	TfdA family taurine dioxygenase
	B8NBZ9	109	TIITLPEK(bz)MSAEK	Cystathionine beta-synthase
	B8NCS4	22	VTK(bz)AAWPYMLK(bz)QK	Peroxisomal multifunctional beta-oxidation protein Fox2
	B8NCS4	30	VTK(bz)AAWPYMLK(bz)QK	Peroxisomal multifunctional beta-oxidation protein Fox2
	B8NFP9	321	KNQHK(bz)LGFMADK	Alcohol dehydrogenase, zinc-containing
	B8NH26	491	LTK(bz)SRNAPK	OTU-like cysteine protease
	B8NIT9	81	QWVNK(bz)LTLEEK	Beta-glucosidase 1
	B8NPB1	27	RK(bz)K(bz)GDK(bz)NNAQAAEQK	KGD domain-containing protein
	B8NPB1	24	RK(bz)K(bz)GDK(bz)NNAQAAEQK	KGD domain-containing protein
	B8NPB1	23	RK(bz)K(bz)GDK(bz)NNAQAAEQK	KGD domain-containing protein
	B8NQ19	110	PQGK(bz)PPK(bz)EDK	Phospholipase D
	B8NQ19	107	PQGK(bz)PPK(bz)EDK	Phospholipase D
	B8NQ62	229	PLLVGNCK(bz)K(bz)R	Homoaconitase
	B8NQ62	230	PLLVGNCK(bz)K(bz)R	Homoaconitase
	B8NSD9	73	DILTK(bz)QGYQVEFLK	D-3-phosphoglycerate dehydrogenase
	B8NUP4	981	FNK(bz)PK(bz)DR	RNA-dependent RNA polymerase
	B8NUP4	979	FNK(bz)PK(bz)DR	RNA-dependent RNA polymerase
	B8NVQ6	26	K(bz)VDFLAGTGNR	Histidinol dehydrogenase
	B8NVW5	6	MLAREK(bz)RYQVTK	Short chain dehydrogenase
	B8NW97	523	TTPK(bz)GTTQR	NRPS-like enzyme

	B8NWU0	165	GHPSGHPLNVGLPQGSIERVIDK(bz)K	UBIQUITIN_CONJUGAT_2 domain-containing protein
	B8NXC8	328	IPK(bz)GTK(bz)IIIPQR	Cytochrome P450
	B8NXC8	325	IPK(bz)GTK(bz)IIIPQR	Cytochrome P450
	B8NRA2	325	EEVRLRAQMQQK(bz)LAEK	Transcriptional regulator Cwf13/SkiP
	B8NVV5	23	HENLK(bz)RHAK	C2H2-type domain-containing protein
	B8NX53	119	EASNTFTK(bz)HGR	BZIP domain-containing protein
	B8NYK9	526	K(bz)SSNNLK(bz)PPNNLK	C2H2 finger domain protein
	B8NYK9	520	K(bz)SSNNLK(bz)PPNNLK	C2H2 finger domain protein
Ribosome biogenesis	B8N3J1	961	VMIKQGIIK(bz)PMEK	Small nucleolar ribonucleoprotein complex subunit Utp14
	B8N4W6	719	RHQK(bz)DRMQK	Urb2 domain-containing protein
	B8NFQ6	94	NLVAATVK(bz)AMERVEEIR	Ribosome biogenesis protein RLP24
Chromatin regulator	B8N5X9	667	VLEVAK(bz)SDIDKR	Nuclear cohesin complex subunit Psc3
	B8NFM9	8	VANPK(bz)HRRSGGASTPHK	Condensin complex subunit 2
	B8NG55	347	VRVYSCK(bz)TLR	ASTRA-associated protein 1
Transporter	B8MZK0	385	EPYMAK(bz)LQPFIQQNKER	GTPase activating protein Sar1
	B8N1A8	170	NTK(bz)LDAQKNR	Calmodulin-binding protein Sha1
	B8NHL1	307	RK(bz)IQLDASDQLFSQLR	Vacuolar sorting protein
Receptor	B8MZL9	289	IVSPNGK(bz)R	Integral plasma membrane protein
	B8NCN8	38	K(bz)VNWALK	Penicillin-binding protein
	B8NFB8	117	RAMLVPSK(bz)QQR	A-pheromone receptor PreA
Translation regulator	B8MZC7	5	K(bz)GGENSKKAAGNARK	DUF1014 domain protein
	B8NLF1	6	GK(bz)EDK(bz)QHINIVVIGHVDSGK	Elongation factor 1-alpha
	B8NLF1	3	GK(bz)EDK(bz)QHINIVVIGHVDSGK	Elongation factor 1-alpha
Catalytic subunit	B8MZP1	8	KPSK(bz)YGNK	DNA polymerase epsilon catalytic subunit
	B8NRT8	956	EAEQRRIK(bz)QAEDER	Histone deacetylase complex subunit Hos4
Kinase	B8NR43	494	LWK(bz)WMAQHLKER	APH domain-containing protein

	B8NC83	7	KPNSDK(bz)R	Protein kinase domain-containing protein
Fermentation	B8NGN6	1507	PMIK(bz)SNFQAR	Fermentation associated protein Csf1
RNA splicing	B8NR51	483	NK(bz)EK(bz)NK(bz)EK	U2AF domain containing protein
	B8NR51	481	NK(bz)EK(bz)NK(bz)EK	U2AF domain containing protein
	B8NR51	485	NK(bz)EK(bz)NK(bz)EK	U2AF domain containing protein
Other	B8NKE0	40	TLEAIATAFLILDSLALK(bz)TLK	Uncharacterized protein

Table S2. Fungal strains and plasmids used in this study.

Strain and plasmid	Description	Reference
A.flavus CA14PTS	Δ*ku70*, Δ*pyrG*	[67]
Wild type	Δ*ku70*, Δ*pyrG*::*AfpyrG*	[32]
Δ*mystA*	Δ*ku70*, Δ*pyrG*, Δ*AflmystA*::*pyrG*	[32]
Δ*mystB*	Δ*ku70*, Δ*pyrG*, Δ*AflmystB*::*pyrG*	[32]
Δ*rtt109*	Δ*ku70*, Δ*pyrG*, Δ*Aflrtt109*::*pyrG*	[30]
Δ*gcnE*	Δ*ku70*, Δ*pyrG*, Δ*AflgcnE*::*pyrG*	[31]
Δ*hatA*	Δ*ku70*, Δ*pyrG*, Δ*AflhatA*::*pyrG*	This study
K321-HA	Δ*ku70*, Δ*pyrG*, Δ*AfladhB*::*AfladhB*::*ha*::*pyrG*	This study
K321R-HA	Δ*ku70*, Δ*pyrG*, Δ*AfladhB*::*AfladhB*^{K321R}::*ha*::*pyrG*	This study
K321A-HA	Δ*ku70*, Δ*pyrG*, Δ*AfladhB*::*AfladhB*^{K321A}::*ha*::*pyrG*	This study
gcnE^{xylP}-*adhB*-HA	Δ*ku70*, Δ*pyrG*, Δ*AflgcnE::pyrG::xylP::AflgcnE*, Δ*AfladhB::AfladhB::ha::pyrG*	This study
Δ*adhB*	Δ*ku70*, Δ*pyrG*, Δ*AfladhB*::*pyrG*	This study
adhB-com	Δ*ku70*, Δ*pyrG*, Δ*AfladhB*::*AfladhB*::*pyrG*	This study
adhB^{K321R}	Δ*ku70*, Δ*pyrG*, *AfladhB*^{K321R}::*pyrG*	This study
adhB^{K321A}	Δ*ku70*, Δ*pyrG*, *AfladhB*^{K321A}::*pyrG*	This study
gcnE^{xylP}	Δ*ku70*, Δ*pyrG*, Δ*AflgcnE::pyrG::xylP::AflgcnE*::*pyrG*	This study
Δ*ada2*	Δ*ku70*, Δ*pyrG*, Δ*Aflada2*::*pyrG*	This study
Δ*ada3*	Δ*ku70*, Δ*pyrG*, Δ*Aflada3*::*pyrG*	This study
gcnE^{xylP}-Δ*ada2*	Δ*ku70*, Δ*pyrG*, Δ*AflgcnE::pyrG::xylP::AflgcnE*, Δ*Aflada2::pyrG*	This study
gcnE^{xylP}-Δ*ada3*	Δ*ku70*, Δ*pyrG*, Δ*AflgcnE::pyrG::xylP::AflgcnE*, Δ*Aflada3::pyrG*	This study
gcnE$^{\Delta BRO}$	Δ*ku70*, Δ*pyrG*, *AflgcnE*$^{\Delta BRO}$::*pyrG*	This study
gcnE$^{\Delta GNAT}$	Δ*ku70*, Δ*pyrG*, *AflgcnE*$^{\Delta GNAT}$::*pyrG*	This study
gcnE^{E139H}	Δ*ku70*, Δ*pyrG*, *AflgcnE*^{E139H}::*pyrG*	This study
Escherichia coli	DH5α	Takara, Japan
Escherichia coli	Rosetta (DE3)	Takara, Japan
pET-32a	pET-32a	This study
pET-32a-GcnE	pET-32a, *AflgcnE*	This study

Table S3. PCR primers used in this study.

Primer	Sequence (5'-3')	Characteristics
Hat-AF	GAGTACAACAGGTCGATGC	To amplify *hatA* upstream fragment
Hat-AR	GGGTGAAGAGCATTGTTTGAGGCTAGGATAGGAGGAAGTAATG	
Hat-BF	GCATCAGTGCCTCCTCTCAGACCTATTCGGTTTCCAGAGTTG	To amplify *hatA* downstream fragment
Hat-BR	TATCCACTTTACCCTCGTTG	
Hat-NF	AGTGAGCCCTGATGTAGTTAG	Nest-primers for fusion PCR
Hat-NR	AGCGAATCTAGCGTTACAG	
ADH-AF	AGGGAAACGGTAGGGTGGA	To amplify *adhB* upstream fragment
ADH-AR	GAAGAGCATTGTTTGAGGCCTTGTAGACTGATCTATGTTGTGGA	
ADH-BF	GCATCAGTGCCTCCTCTCAGACGTGCGGACCCAATGAAGG	To amplify *adhB* downstream fragment
ADH-BR	TGATACGACGCCACCCATAC	
ADH-321KA-AF2	CAACATGCACTAGGGTGGGTC	To amplify K321A mutant site fragment
ADH-321KA-AR1	GACCCACCCTAGTGCATGTTG	
ADH-321KR-AF2	CAACATCGACTAGGGTGGGTC	To amplify K321R mutant site fragment
ADH-321KR-AR1	GACCCACCCTAGTCGATGTTG	
ADH-321-A2R	GAAGAGCATTGTTTGAGGCTTATTCTCCGGCTTTGAAAATCAC	To amplify K321 mutant upstream fragment
ADH-NF	GTGCGTCCTTAAGAGGCGTAC	Nest-primers for fusion PCR
ADH-NR	GGAGGTTGCGTGGAAATAGAG	
ADH-HA-AR	ACCACTACCTCCGCCACCTTCTCCGGCTTTGAAAATCAC	To amplify HA upstream fragment
TestF	GCCCCCAATCGTCAAGGTTT	To identification *pyrG* insertion
TestR	AGAGCGCCTTGAGAAAACCAGA	
gcne-AF	CTTTGTCTTATAGCACCTGGC	To amplify *gcnE*^{xylP} upstream fragment
gcne-AR	GGGTGAAGAGCATTGTTTGAGGCATGTCGAATAGTGAGATCGGTC	
gcne-BF	CATTCATCGACTCGAAGAACCAACATGGCGTCAGAAAGTGAGTGG	To amplify *gcnE*^{xylP} downstream fragment
gcne-BR	GATAGCGGAAATGATCGAAAGG	
gcne-NF	GCCGTTAGTTTGGGCTTAG	Nest-primers for fusion PCR
gcne-NR	TTGTGTTCCCGATACCGAC	
gcne-AF1	CATTATGTAGCCAGGCAGAGC	To amplify *gcnE* domain upstream fragment
GNAT-A1R	CTTGGCGTGTACTGCTTCTTTCTCCCCTCGACGTTCTTCTAG	
BRO-A1R	GAAGAGCATTGTTTGAGGCTCATGTACTGATTGCCGGGATACTTAACG	
GNAT-A2F	CTAGAAGAACGTCGAGGGGAGAAAGAAGCAGTACACGCCAAG	To amplify *gcnE* domain downstream fragment

BRO-A2F	CGTTAAGTATCCCGGCAATCAGTACA TGAGCCTCAAACAATGCTCTTC	
gcne-AR2	GGGTGAAGAGCATTGTTTGAGGCTCA TGTACTTACCGACCACTCG	
gcne-E139H-BF	GCATCAGTGCCTCCTCTCAGACGTGC AGCCCTGGTTTGGTGA	To amplify *gcnE* point mutant downstream fragment
gcne-E139H-BR	GGAAAGAACGTCTAGGTCGGG	
gcne-E139H-AF2	GCAGATTTGCGCACATTGTC	To amplify E139H mutant site fragment
gcne-E139H-AR1	CAAAAGACAATGTGCGCAAATC	
gcne-139Q-NF1	GGAAACAGCAGAAAAGATGGG	Nest-primers for fusion PCR
gcne-139Q-NR1	CTGGCGAATGAGTAAAGTAACG	
gcne-D-AR	GCTTCCGTCGACCTCGAGAGGTTATA ACGCTTGCTTGTCAG	To amplify *gcnE* deletion upstream fragment
gcne-D-BF	CTGACAAGCAAGCGTTATAACCTCTC GAGGTCGACGGAAGC	To amplify *gcnE* deletion downstream fragment
ada2-AF	CCAAGACATTGTGACAGGAGG	To amplify *ada2* upstream fragment
ada2-AR	GGGTGAAGAGCATTGTTTGAGGCCGA TTGTGAATCCCTTTCATC	
ada2-BF	GCATCAGTGCCTCCTCTCAGACGTCG ACAGTTTTATATCCAGGG	To amplify *ada2* downstream fragment
ada2-BR	CGTTATGGATGAATGGATGG	
ada2-NF	GGGTTGGTGCAGTGACAGAG	Nest-primers for fusion PCR
ada2-NR	GCTAGTCTGCGATACGTAACGG	
ada3-AF	CTGGACCTGGAAAGAGCGTG	To amplify *ada3* upstream fragment
ada3-AR	GGGTGAAGAGCATTGTTTGAGGCATG ACGGCCCGAAAAGGTG	
ada3-BF	GCATCAGTGCCTCCTCTCAGACCTTC GCTTGCTCCTCATGC	To amplify *ada3* downstream fragment
ada3-BR	CAACGAGTATGCCTCCCATAAC	
ada3-NF	GACGAGCCATTTACATACCC	Nest-primers for fusion PCR
ada3-NR	GCTCTGGTACAGTTTTGACATG	
ADH-32aF	CGGGATCCATGAGCACGGAAAACAA GATGC	To amplify *adhB*-pET-32a fragment
ADH-32aR	CCCAAGCTTTTATTCTCCGGCTTTGAA AATCA	
ADH^{K321A}-32aF	CCAACATGCACTAGGATTCATGGCGG ACAAGA	To amplify *adhB*^{K321A}-pET-32a fragment
ADH^{K321A}-32aR	ATCCTAGTGCATGTTGGTTCTTTTTCA AGACATCC	
ADH^{K321R}-32aF	CCAACATCGACTAGGATTCATGGCGG ACAAGA	To amplify *adhB*^{K321R}-pET-32a fragment
ADH^{K321R}-32aR	ATCCTAGTCGATGTTGGTTCTTTTTCA AGACATCC	

Gcne-32aF	GCCATGGCTGATATCGGATCCCTGGAAGTTCTGTTCCAGGGGCCCATGGCGTCAGAAAACAACAAAC	To amplify *gcne*-pET-32a fragment
Gcne-32aR	GGTGGTGGTGGTGGTGCTCGAGTCATGTACTTACCGACCACTCG	

Table S4. qPCR primers used in this study.

Primer	Sequence (5’-3’)	Characteristics
hat-QF	GAAGACCCTAATGAAGCGTTTGAC	To detect the *hatA* gene transcript level
hat-QR	GAGGCAATCTTATAGGTGGAACG	
adh-QF	CAATGCCCTGGAGTTCAAGC	To detect the *adhB* gene transcript level
adh-QR	CAAAGCCTGTCCTCAGTGCC	
brlA-QF	GCCTCCAGCGTCAACCTTC	To detect the *brlA* gene transcript level
brlA-QR	TCTCTTCAAATGCTCTTGCCTC	
abaA-QF	CACGGAAATCGCCAAAGAC	To detect the *abaA* gene transcript level
abaA-QR	TGCCGGAATTGCCAAAG	
nsdC-QF	GCCAGACTTGCCAATCAC	To detect the *nsdC* gene transcript level
nsdC-QR	CATCCACCTTGCCCTTTA	
sclR-QF	CAATGAGCCTATGGGAGTGG	To detect the *sclR* gene transcript level
sclR-QR	ATCTTCGCCCGAGTGGTT	
aflR-QF	AAAGCACCCTGTCTTCCCTAAC	To detect the *aflR* gene transcript level
aflR-QR	GAAGAGGTGGGTCAGTGTTTGTAG	
aflS-QF	CGAGTCGCTCAGGCGCTCAA	To detect the *aflS* gene transcript level
aflS-QR	GCTCAGACTGACCGCCGCTC	
aflM-QF	ATCCTGACCAGCTCTAACAC	To detect the *aflM* gene transcript level
aflM-QR	ATCTTCTTGTCTCCGCAGTC	
aflN-QF	TTCATTCCTGAGCGATGG	To detect the *aflN* gene transcript level
aflN-QR	CGTATGCTGGCGTAATATC	
aflQ-QF	GTCGCATATGCCCCGGTCGG	To detect the *aflQ* gene transcript level
aflQ-QR	GGCAACCAGTCGGGTTCCGG	
aldA-QF	TGGCTGGGCCGATAAGATT	To detect the *aldA* gene transcript level
aldA-QR	GTGGCGGGTGTAGGTAAGAGACT	
facA-QF	CGCAAGTCGATTGGACCATT	To detect the *facA* gene transcript level
facA-QR	ATCTTACCGCTGCGAGTCTTAGG	
fksA-QF	ACAACATGCCCACCTTCACAG	To detect the *fksA* gene transcript level
fksA-QR	GCTCTTGGCGACATCCTTCTC	
chsB-QF	CTGATCGCGAAAACTGGAGG	To detect the *chsB* gene transcript level
chsB-QR	CATGTGACGTCGTGTGTGTT	
chsD-QF	TTGGTGCGAGACTTGTGCG	To detect the *chsD* gene transcript level
chsD-QR	ACGTAGAAGGTAAATGCGATAGCTG	
ITS2-QF	CACGGCTTGTGTGTTGGGTC	To detect the *its2* gene transcript level
ITS2-QR	CCTACAGAGCGGGTGACAAAG	
ada2-QF	GCACAATCTACGGTTAGCCATC	To detect the *ada2* gene transcript level
ada2-QR	ACTTCGTCTTGGCTGGTTGC	
ada3-QF	GGAAGAGGCACTACAACGAG	To detect the *ada3* gene transcript level
ada3-QR	CGGTGTTCGTATCGTAGTAGAG	
gcnE-QF	GAGAGGGAACCAAAGTCTGAAGG	

gcnE-QR	GGAACAATCCTCAATGGTGGC	To detect the *gcnE* gene transcript level
actin-QF	ACGGTGTCGTCACAAACTGG	To detect the *actin* gene transcript level
actin-QR	CGGTTGGACTTAGGGTTGATAG	
